# Stalled oligodendrocyte differentiation in IDH-mutant gliomas

**DOI:** 10.1186/s13073-023-01175-6

**Published:** 2023-04-13

**Authors:** Yanfei Wei, Guanzhang Li, Jing Feng, Fan Wu, Zheng Zhao, Zhaoshi Bao, Wei Zhang, Xiaodong Su, Jiuyi Li, Xueling Qi, Zejun Duan, Yunqiu Zhang, Sandra Ferreyra Vega, Asgeir Store Jakola, Yingyu Sun, Helena Carén, Tao Jiang, Xiaolong Fan

**Affiliations:** 1 Department of Biology, Beijing Key Laboratory of Gene Resource and Molecular Development, School of Life Sciences, and Key Laboratory of Cell Proliferation and Regulation Biology, Ministry of Education, School of Life Sciences, Beijing Normal University, Beijing, China; 2 Beijing Neurosurgical Institute, Beijing, 100070 China; 3 Department of Neurosurgery, Beijing Tiantan Hospital, Capital Medical University, Beijing, 100070 China; 4 Biodynamic Optical Imaging Center (BIOPIC), School of Life Sciences, Peking University, Beijing, 100871 China; 5 College of Life Sciences, Sichuan Normal University, Chengdu, 610101 China; 6 Department of Pathology, San Bo Brain Hospital, Capital Medical University, Beijing, 100093 China; 7 Center of Growth Metabolism & Aging, Key Laboratory of Bio-Resource and Eco-Environment of Ministry of Education, College of Life Sciences, Sichuan University, Chengdu, 610065 China; 8 Department of Clinical Neuroscience, Institute of Neuroscience and Physiology, Sahlgrenska Academy, University of Gothenburg, Gothenburg, 41390 Sweden; 9 Sahlgrenska Center for Cancer Research, Department of Laboratory Medicine, Institute of Biomedicine, Sahlgrenska Academy, University of Gothenburg, 41390 Gothenburg, Sweden; 10 Department of Neurosurgery, Sahlgrenska University Hospital, Gothenburg, 41390 Sweden; 11 Chinese Glioma Genome Atlas Network (CGGA), Beijing, 100070 China

## Abstract

**Background:**

Roughly 50% of adult gliomas harbor *isocitrate dehydrogenase* (*IDH*) mutations. According to the 2021 WHO classification guideline, these gliomas are diagnosed as astrocytomas, harboring no 1p19q co-deletion, or oligodendrogliomas, harboring 1p19q co-deletion. Recent studies report that IDH-mutant gliomas share a common developmental hierarchy. However, the neural lineages and differentiation stages in IDH-mutant gliomas remain inadequately characterized.

**Methods:**

Using bulk transcriptomes and single-cell transcriptomes, we identified genes enriched in IDH-mutant gliomas with or without 1p19q co-deletion, we also assessed the expression pattern of stage-specific signatures and key regulators of oligodendrocyte lineage differentiation. We compared the expression of oligodendrocyte lineage stage-specific markers between quiescent and proliferating malignant single cells. The gene expression profiles were validated using RNAscope analysis and myelin staining and were further substantiated using data of DNA methylation and single-cell ATAC-seq. As a control, we assessed the expression pattern of astrocyte lineage markers.

**Results:**

Genes concordantly enriched in both subtypes of IDH-mutant gliomas are upregulated in oligodendrocyte progenitor cells (OPC). Signatures of early stages of oligodendrocyte lineage and key regulators of OPC specification and maintenance are enriched in all IDH-mutant gliomas. In contrast, signature of myelin-forming oligodendrocytes, myelination regulators, and myelin components are significantly down-regulated or absent in IDH-mutant gliomas. Further, single-cell transcriptomes of IDH-mutant gliomas are similar to OPC and differentiation-committed oligodendrocyte progenitors, but not to myelinating oligodendrocyte. Most IDH-mutant glioma cells are quiescent; quiescent cells and proliferating cells resemble the same differentiation stage of oligodendrocyte lineage. Mirroring the gene expression profiles along the oligodendrocyte lineage, analyses of DNA methylation and single-cell ATAC-seq data demonstrate that genes of myelination regulators and myelin components are hypermethylated and show inaccessible chromatin status, whereas regulators of OPC specification and maintenance are hypomethylated and show open chromatin status. Markers of astrocyte precursors are not enriched in IDH-mutant gliomas.

**Conclusions:**

Our studies show that despite differences in clinical manifestation and genomic alterations, all IDH-mutant gliomas resemble early stages of oligodendrocyte lineage and are stalled in oligodendrocyte differentiation due to blocked myelination program. These findings provide a framework to accommodate biological features and therapy development for IDH-mutant gliomas.

**Supplementary Information:**

The online version contains supplementary material available at 10.1186/s13073-023-01175-6.

## Background

Gliomas are the most common primary tumors in the adult central nervous system [1]. Prognosis for the majority of glioma patients has been only marginally improved over the last decades [2, 3]. On the basis of integrated histomolecular classification scheme, adult diffuse gliomas are currently diagnosed into three main categories: isocitrate dehydrogenase (IDH)-mutant astrocytoma (harboring no 1p19q co-deletion) or oligodendroglioma (harboring 1p19q co-deletion) and IDH-wild-type glioblastoma (GBM) [4, 5]. This subtyping greatly improves objectiveness and risk stratification [6]. However, the putative cells of origin (COO) and presumed levels of differentiation in histological subtypes represent major roadblocks in studies of glioma pathogenesis. Unbiased molecular classification schemes have been proposed on the basis of prognosis [7], co-occurrence and mutual exclusivity of genomic anomalies [6, 8, 9], global transcriptome [10–13], or methylome characteristics [12, 14, 15]. Though these classification schemes are prognostically relevant, subtype-specific therapeutic targets remain unidentified. Mapping gliomas into brain development programs may generate a platform for identification of subtype-specific therapeutic vulnerabilities.

Tumor initiation depends on the interplay between driving genomic alterations and susceptible COO in a seed versus soil manner [16]. We hypothesized that pathways associated with subtype-specific glioma pathogenesis, which are also conserved during normal brain development, may permit an ontology-based molecular classification of glioma [17, 18]. We previously identified reciprocally expressed gene modules consistently co-expressed with EGFR or PDGFRA (named EM or PM, respectively) in glioma transcriptome. Whereas EM genes contain key regulators involved in the initiation of gliogenesis: NFIA, SOX9, and POU3F2 (BRN2) [19–22]; PM genes contain key regulators for specification and maintenance of oligodendrocyte progenitor cell (OPC), including CHD7, MYT1, OLIG1, OLIG2, PDGFRA, SOX4, SOX6, and SOX8 [23–26]. Adult diffuse gliomas of WHO grades 2 to 4 can be robustly assigned into the EM or PM subtype in a histological subtype- and grade-independent manner [17, 18]. The EM subtype corresponds to IDH-wild-type GBM, typically harbors gain of chromosome 7 and loss of chromosome 10 and occurs predominantly in the elderly population with a prognosis shorter than 2 years. The PM subtype contains IDH-mutant astrocytoma and oligodendroglioma, occurs in younger adults, and has better prognosis [17, 18]. To delineate the aberrant developmental program in the PM gliomas, we performed an integrated analysis combining features of bulk transcriptome, single-cell RNA-seq, RNAscope, DNA methylome, and single-cell ATAC-seq on clinical samples from multiple cohorts derived from three continents. We show that PM gliomas are blocked at the premyelinating stage potentially caused by IDH mutation-induced hypermethylation in essential regulators of the myelination program and in myelin components, suggesting suppressed myelination program as a potential therapeutic target for IDH-mutant gliomas. Our findings constitute a framework to accommodate the biological features and to facilitate treatment development against glioma.

## Methods

### Samples and datasets

Fresh tumor samples used for bulk RNA-seq (*n* = 182) or single-cell RNA-seq (*n* = 16) were obtained from Beijing Tiantan Hospital. Archived formalin-fixed paraffin-embedded (FFPE) samples (*n* = 10) used for RNAscope analysis were obtained from Sanbo Brain Hospital, Capital Medical University in Beijing. FFPE samples for DNA methylation analysis (*n* = 106) were obtained from Sahlgrenska University Hospital in Gothenburg, Sweden. Bulk data of glioma transcriptome (*n* = 702) and methylome data (*n* = 385) were downloaded from The Cancer Genome Atlas (TCGA, https://www.cancer.gov/tcga) [12]. Bulk transcriptomes from PM gliomas from CGGA (*n* = 162) [27], Rembrandt (*n* = 253) [28], and GSE4290 (*n* = 92) [29] datasets, analyzed in our previous studies [17, 18], were used for analyzing genes differentially expressed between PM and NT brain tissues and the expression profiles of oligodendrocyte lineage stage-specific markers or signatures in PM gliomas. Transcriptome data from murine neural cell types (GSE52564 (*n* = 17) [30]) and human brain development (GSE25219 (*n* = 15 stages) [31]), downloaded from Gene Expression Omnibus (GEO, https://www.ncbi.nlm.nih.gov/geo/), were used for the analysis of conserved expression of PM glioma-specific genes during brain development. Single-cell transcriptomes of glioma samples (GSE70630 for IDH-mutant oligodendroglioma (*n* = 4) [32], GSE89567 for IDH-mutant astrocytoma (*n* = 9), [33] and GSE131928 for IDH-wild-type GBM (*n* = 7) [34]), downloaded from GEO, were used for the analysis of cell populations and the expression of the markers of glial lineage and differentiation stages. Single-cell ATAC-seq data from IDH-mutant gliomas (*n* = 9), downloaded from the European Genome-phenome Archive repository under EGAS00001004523 [35], were used for the analysis of chromatin status of key members of myelination program and OPC specification and maintenance in PM gliomas.

### Analysis of bulk transcriptome data

Qlucore Omics Explorer 3.6 (Qlucore AB, Lund, Sweden) was used to analyze the bulk transcriptome data. Transcriptomes of the PM gliomas with or without 1p19q co-deletion were compared with the transcriptomes of non-tumor (NT) brain tissues using *t*-test, and the top 2000 most differentially expressed probe sets for Rembrandt and GSE4290 dataset and top 1000 genes for CGGA dataset were detected, respectively. Genes concordantly up-regulated in both PM glioma subtypes were identified and analyzed in functional enrichment analysis tool (Funrich) software [36, 37] for the generation of the Venn diagrams, and GO terms were defined by using DAVID functional annotation bioinformatics microarray analysis (https://david.ncifcrf.gov/) [38, 39]. The expression pattern of PM glioma-enriched genes in oligodendrocyte lineage differentiation was analyzed in dataset GSE52564 [30]. Scatter plot analyses were performed to compare the expression of oligodendrocyte lineage stage-specific markers and regulators, as well as markers of astrocyte precursor cell (APC) and mature astrocyte, except for the PM genes and OPC markers reported in our previous studies [17, 18], between the PM gliomas and NT brain tissues.

### Transcriptome-based prediction of 1p19q co-deletion

Based on the 100 K SNP data in the REMBRANDT dataset, the status of 1p19q co-deletion in PM gliomas was previously determined [17]. The transcriptomes between 34 PM gliomas with 1p19q co-deletion and 62 PM gliomas without 1p19q co-deletion were compared with *t*-test at *p* = 1e − 16 and *q* = 2.708e − 14, generating a classifier of 152 differentially expressed genes between the two PM glioma subtypes (named 1p19q classifier). The performance of this 1p19q classifier was validated using glioma samples from TCGA. Out of 703 TCGA samples, 350 were classified as PM gliomas using the EM/PM signature. Using our 1p19q classifier, 140 and 210 PM gliomas were predicted as harboring and not harboring 1p19q co-deletion, respectively. Hundred and thirty-three of the 138 samples predicted with 1p19q co-deletion and 202 of 202 samples predicted with 1p19q non co-deletion were validated using SNP6.0 data, demonstrating a high fidelity of the 1p19q classifier.

To predict 1p19q co-deletion status in PM gliomas from GSE4290 and in the REMBRANDT dataset without annotation of 1p19q co-deletion, the EM/PM clustering was first performed [17], the PM gliomas were subsequently clustered using the 1p19q classifier.

### Composition of the 1p19q classifier

1p19q classifier contains the following genes:

ABCC8 ACTL6B ADPRHL2 AK2 AKIRIN1 ALDH1L2 ANO5 ATCAY ATOH8 CACNG2 CAP1 CAPNS1 CAPZA1 CAPZB CARD8 CCDC23 CCNL2 CDC42 CDHR1 CHGB CLVS1 CMBL CMPK1 CRTAC1 CSDE1 CYB561D1 DDX20 DNAJC8 DOCK7 DR1 EID2 ERCC1 EXOSC10 EXTL2 EYA3 FAM155A FBXO42 FKRP FNBP1L FOXJ3 FPGT GABRB3 GDAP1L1 GFRA1 GNAI3 GNB1 GNG12 GNG5 GPBP1L1 GRIN3A HDAC1 HEXIM2 HIP1R HMGCL HP1BP3 HSPB11 IQGAP1 IRF3 KCNIP2 KCNK3 KDELR1 KIAA0355 KIAA2013 KPNA6 L1CAM LRPPRC LRRC41 LRRC8D LRRTM4 LSM10 LSM14A MCPH1 MEAF6 MFN2 MIER1 MMAB MRPS15 MTF1 MTF2 NADK NDUFA3 NECAP2 NOG NRAS NRD1 NUDT7 PDE8A PEF1 PHACTR4 PHF13 PKN2 PLEKHM2 POMGNT1 PPCS PPP1R8 PRPF31 PTP4A2 RABAC1 RAP1A RER1 REST RPF1 RPL22L1 S100PBP SCAMP1 SCMH1 SCP2 SDF4 SEZ6L SEZ6L2 SH3GLB1 SHISA9 SLC30A7 SLC8A3 SPR SRRM1 SRSF11 SRSF4 SSU72 STK40 SVOP TCEB3 TMEM167B TRAPPC3 TRIM67 TRIT1 TXNDC12 UBA2 UROD WASF2 YTHDF2 ZBTB8OS ZCCHC11 ZCCHC17 ZDHHC22 ZNF45 ZNF518B ZZZ3 C1orf144 C1orf151 C1orf175 C2orf67 FAM123C FAM54B HBXIP KIAA0485 KIAA1409 PNRC2 LOC257396 LOC283484 MYH6 NCRNA00219.

### Co-staining of myelin structure and IDH1 mutation

Sections of 5 µM from FFPE glioma samples were baked at 72℃ for 30 min, then deparaffinized, rehydrated, and treated in 10 mM citrate buffer (100℃, 10 min) for antigen repair. Subsequently, the sections were immersed in ethanol containing 3% hydrogen peroxidase for 10 min to block endogenous peroxidase activity. The sections were incubated overnight at 4℃ with anti IDH1 R132H mAb (Dianova, clone H09, lot: 161,122/16 at 1: 500 dilution). Following twice 5 min washing in PBS, the sections were incubation with peroxidase-conjugated secondary anti-mouse antibodies (Beijing Zhongshan Jinqiao Biotechnology, lot: WP20062902), washed with PBS, stained with 3,3’-diaminobenzidine. Subsequently, the sections were hydrated in 95% ethanol for 1 min, stained with laxol fast blue (LFB) staining solution (Solvent Blue 38, Sigma S3382, lot: 072K1363; 1 g solvent blue dissolved in 100 ml of 95% ethanol and 0.5 ml 10% acetic acid) at 60 °C for 1 h. Following cooling to room temperature, the sections were rinsed in 95% ethanol for 30 s to remove excess of dye and then rinsed in distilled water. The slides were differentiated by dipping in 0.5% lithium carbonate solution for 10 s, then rinsed in distilled water and stained with hematoxylin for 1 min. Following hematoxylin staining, the sections were rinsed thoroughly, further differentiated in hydrochloric acid alcohol for 10 s, rinsed and incubated with ammonia solution for 1 min. Finally, the sections were rinsed and dehydrate in 70%, 95%, and 100% ethanol, cleaned with xylene, and mounted with cover slide.

### Single-cell RNA-seq

Fresh tumor samples were collected at the time of resection from the operating room. Following washing with phosphate-buffered saline (PBS), the samples were minced with a surgical scissors, then enzymatically dissociated using trypsin–EDTA solution. Following filtrating through a cell strainer and red blood cell lysis, the cell suspension was loaded into chromium microfluidic chips with 30 chemistry and barcoded with a 10X Chromium Controller (10X Genomics). RNA from the barcoded cells was subsequently reverse-transcribed. Sequencing was performed according to 10X Genomics recommended single-cell RNA-seq protocol on the Illumina NovaSeq 6000 with a paired-end 150 bp reading strategy.

### Single-cell RNA-seq data processing

The Read10X function in Seurat (version 3.1.0) (https://satijalab.org/seurat/vignettes.html) [40, 41] generated the reads in the output of the cellranger pipeline from 10X, which returns a unique molecular identified (UMI) count matrix. Cells in the UMI count matrix with fewer than 200 transcripts and genes with fewer than three cells were removed. The matrix was then analyzed with default parameters in Seurat. The workflow included the following steps: data pre-processing and normalization, feature selection (nfeatures = 500), data scaling, linear dimensional reduction, determination of the dimensionality for principal components with *p* values < e − 10, and *t*-SNE-based cell clustering and identification of cluster markers. Cell populations were assigned based on canonical markers of neural cells and tumor microenvironment as listed below:

- Pre-OPC: ASCL1, BTG2, HES6, DLL1, DLL3 [42].
- OPC: PDGFRA, CSPG4, PCDH15, PTPRZ1 [43].
- COP: NEU4, SOX6, VCAN [43].
- Astrocyte: ALDOC, AQP4, CLU, GFAP [44], MLC1 [30], S100β and GLT1 [21, 45–48].
- APC: FABP7, FGFR3 and GLAST [21, 45–48].
- Oligodendrocyte: MOBP, MBP, MOG [32], Klk6 [43].
- T cell: CD2, CD3D, CD3E, CD3G [34].
- Microglia: CX3CR1, P2RY12 [33], TMEM119 [49].
- Macrophage: CD163 [33], S100A8, S100A9 [49], AIF1 [50].
- Cell proliferation: MKI67, TOP2A, CDK1 [51].

### CNV analysis in single cell RNA-seq data

InferCNV (R package, https://github.com/broadinstitute/inferCNV) was used to analyze single-cell RNA-Seq data for inferring gains or deletions of entire chromosomes or large chromosomal segments. We used non-malignant cells (microglia/macrophages or oligodendrocytes) as reference cells. Cutoff parameters at 1.0, 1.0, and 0.1 were used for the data derived from STRT-seq, Smart–seq2, and 10X Genomics platforms, respectively. Other parameters were by default.

### Canonical correlation analysis (CCA) between glioma cells and cell populations in murine oligodendrocyte lineage

To correlate cell types identified in PM gliomas to the transcriptomes of normal cells in murine oligodendrocyte lineage differentiation, we first extracted gene expression data of OPC, COP, and MOL from previously published dataset [43], transcriptomes of human glioma cells and murine oligodendrocyte lineage cells were then combined and normalized using the Seurat program, and the Pearson correlation coefficient values were calculated using “cor” function in R.

### RNAscope analysis

Sections of 5 µm thickness from FFPE samples were obtained from the glioma biobank at Sanbo Brain Hospital, Capital Medical University in Beijing. RNAscope Multiplex Fluorescent Reagent Kit v2 (Advanced Cell Diagnostics, cat. no. 323100) was used. Slides were baked for 1 h at 60 °C, deparaffinized, and dehydrated with xylene and ethanol. The sections were then pretreated with RNAscope hydrogen peroxide for 10 min at room temperature and RNAscope target retrieval reagent (1 ×) for 15 min at 98 °C. RNAscope protease plus was then applied to the sections for 30 min at 40 °C. The sections were subsequently hybridized with mixed probes (pre-heated to 40 °C) from Advanced Cell Technologies targeting PCDH15 (an OPC marker, C3 channel, cat. no. 525881), VCAN (a COP marker, C2 channel, cat. no. 430071), and ALDOC (an astrocytic marker, C1 channel, cat. no. 407031) for 2 h at 40 °C. Finally, amplification steps were performed with the reagents provided in the RNAscope Multiplex Fluorescent Reagent Kit v2. Following an incubation with DAPI for 30 s at room temperature, the sections were sealed with Prolong Gold (cat. no. P36930) anti-quench reagent. The hybridization results were imaged using an inverted Zeiss LSM700 confocal microscope and analyzed using ZEN 2009 Light Edition software.

### DNA methylation analysis

DNA methylomes from 385 TCGA samples including 109 and 173 IDH-mutant WHO grade 2–3 gliomas with or without 1p19q co-deletion, respectively, 103 IDH wild-type EM gliomas [17], and 106 IDH-mutant gliomas from the Sahlgrenska University Hospital from Gothenburg, Sweden [52] were analyzed. The generation of DNA methylome data from FFPE samples is recently reported [52]. DNA methylomes from 276 normal brain samples (GSE43414 [53]) served as control. Two samples from the TCGA data were excluded due to poor data quality. DNA methylation data (iDAT files) derived from HumanMethylation450 BeadChip were processed using the R package watermelon and normalized with dasen. We identified differentially methylated positions (DMPs) and differentially methylated regions (DMR) using the R package ChAMP [54]. Bumphunter was used for DMR analysis. Gene plots were generated using the Gviz [55] in R package.

### Analysis of single-cell ATAC-seq data

Single-cell ATAC-seq data were analyzed by using ArchR package [56]. QC filtering was performed, cells with at least 1000 unique nuclear fragments per cell, and a TSS enrichment score greater than 0.5 were retained. We next performed dimensionality reduction using Iterative Latent Semantic Indexing (LSI) and clustering using a graph-based approach implemented in Seurat with the FindClusters function, and harmony was used for batch effect correction between the samples. We assigned clusters by using canonical signature genes of malignant or non-malignant cell populations described above and identified marker features of each cluster. Finally, we browsed the local chromatin accessibility of these marker genes on each cluster based on genome browser tracks.

### Statistical analysis

For statistical analyses, mean and standard deviation (SD) of the results were shown in dot plots. One-way ANOVA test was performed for comparisons between more than two groups. The statistical analyses were performed using GraphPad Prism 7/8 software, and statistical significances at *p* < 0.05, *p* < 0.01, *p* < 0.001, and *p* < 0.0001 were labeled as *, **, ***, and ****, respectively.

## Results

### Bulk transcriptome-based identification of differentiation blockage in PM gliomas

**Fig. 1:**
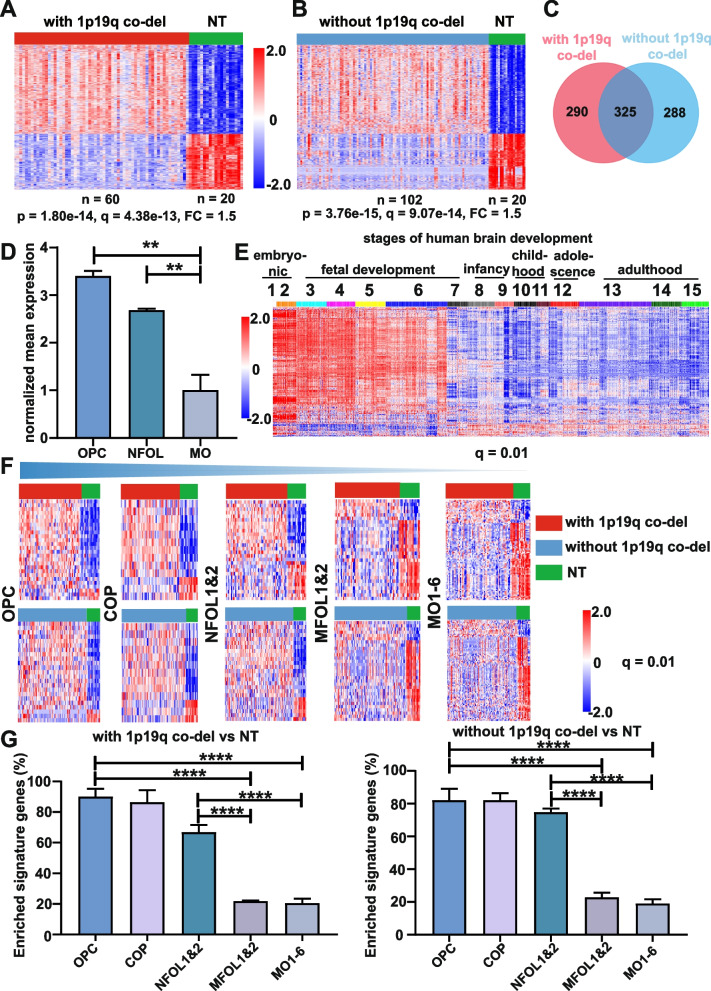
Declining expression of NFOL and MFOL signatures in PM gliomas from the CGGA dataset. **A**, **B** Heatmaps of differential gene expression profile between PM gliomas and NT brain tissues at the indicated *p* and *q* values with a fold change (FC) at 1.5. Genes concordantly enriched in both PM glioma subtypes (**C**) show upregulated expression in OPC and NFOL of mouse oligodendrocyte lineage as analyzed in GSE52564 [30] (**D**), and in early stages of human brain development as analyzed in GSE25219 [31] (**E**). **F** Drastically declining expression in the gene expression signatures of NFOL and MFOL in PM gliomas with (upper) or without (lower) 1p19q co-deletion. The same color codes were used in **A**, **B**,** C**, and **F**. Data shown in **A** to **F** were derived from CGGA dataset. **G** Percentages of the members of the indicated stage-specific signature gene sets enriched in PM gliomas from CGGA, GSE4290, and Rembrandt datasets; mean and standard deviation are shown. NT: non-tumor brain tissues, OPC: oligodendrocyte progenitor cell, COP: differentiation-committed oligodendrocyte precursors, NFOL: newly formed oligodendrocyte, MFOL: myeline-forming oligodendrocyte, MO: mature oligodendrocyte. **p < 0.01; ****p < 0.0001

Ninety-six percent PM gliomas from the TCGA cohort harbored mutations in *IDH1/2*. In agreement with previous reports [8, 9], PM gliomas were further divided into two subtypes based on mutually exclusive sets of genomic alterations. Mutations in *CIC* and *TERT* promoter were found in 46% and 43% PM gliomas with 1p19q co-deletion, respectively, while mutations in *TP53* and *ATRX* in 88% and 74% PM gliomas without 1p19q co-deletion, respectively (Additional file 1: Fig. S1). In the four datasets analyzed, PM gliomas accounted for 53.4% of all 1389 adult gliomas, both subtypes contained all histological subtypes and grades (Additional file 2: Table S1). Using non-tumor (NT) brain samples as the control, we identified genes concordantly enriched in both PM glioma subtypes in the CGGA [27], GSE4290 [29], and REMBRANDT [28] datasets (Fig. 1A–C, Additional file 1: Fig. S2A-C and Fig. S3A-C). Along the differentiation hierarchy of oligodendrocyte lineage, these genes were enriched in OPCs, their expression progressively declined in premyelinating newly formed oligodendrocytes (NFOL) and mature oligodendrocytes (MO) (Fig. 1D, Additional file 1: Fig. S2D, Fig. S3D and Fig. S4). However, these genes were not differentially expressed between developing astrocyte and mature astrocyte (Additional file 1: Fig. S5). During human brain development, these genes were upregulated in early stages (periods 1 to 6, between 4 and 24 post-conceptual weeks) (Fig. 1E and Additional file 1: Fig. S2E and Fig. S3E). The products of these up-regulated genes are involved in numerous diverse biological functions (Additional file 1: Fig. S6). We also assessed the expression pattern of gene sets characteristic of the oligodendrocyte differentiation stages [30, 43] in PM gliomas. In all three datasets analyzed, the heatmaps and the percentages of the members of the respective signature gene set enriched in PM gliomas show that PM gliomas were enriched in the signatures of OPC and differentiation-committed oligodendrocyte precursor (COP). A drastic decline in the expression between the signatures of NFOL to myelin-forming oligodendrocyte (MFOL) was reproducibly observed (Fig. 1F and G and Additional file 1: Fig. S2F and Fig. S3F). The findings together suggest a blockage at the transition between NFOLs and MFOL in both subtypes of PM gliomas. Notably, in the absence of NT controls, most differentially expressed genes between PM gliomas with or without 1p19q co-deletion manifested the effects of 1p19q co-deletion, mutations in *CIC* or *TP53*, and the activities of tumor microenvironment (Additional file 1: Fig. S1).

**Fig. 2:**
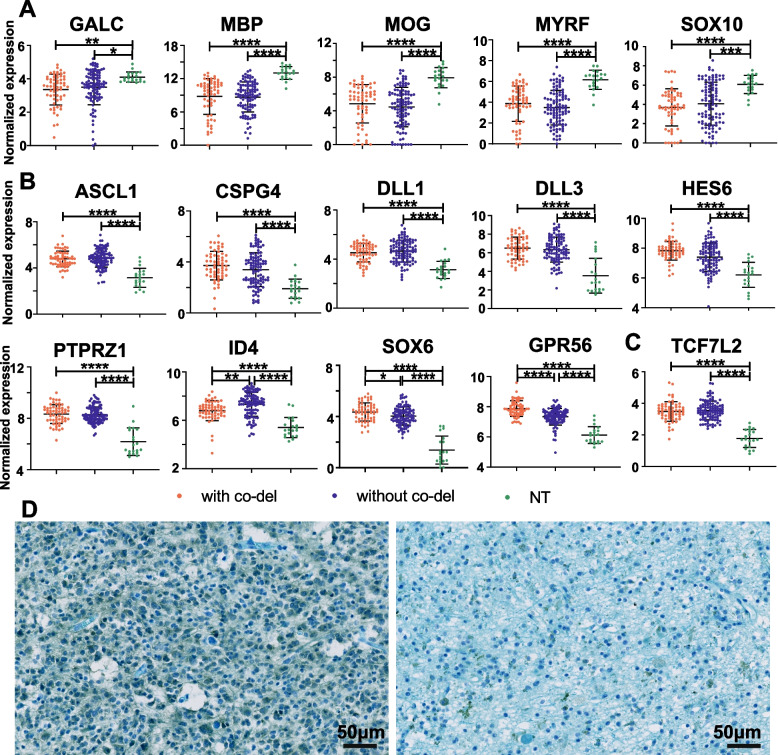
Suppressed myelination program in PM gliomas from the CGGA dataset. **A** Under expression in PM gliomas of the MO marker GALC/O1, myelin components (MBP, MOG), and myelination regulators MYRF/C11ORF9. **B** Sustained expression of stage-specific markers of pre-OPC and OPC in PM gliomas. **C** Sustained expression of stage-specific marker of NFOL in PM gliomas. **D** Co-staining with LFB which labels myelin structures, and with the anti-IDH1 R132H antibody which stains IDH1 mutant cells, of a representative PM glioma with 1p19q co-deletion is shown for tumor (left) and NT (right) regions. Myelin fiber is absent in tumor regions with strong cytosolic staining of the IDH1 R132H mutant protein

We next compared the expression of canonical markers and regulators sequentially expressed in oligodendrocyte lineage in PM gliomas and in NT controls [23, 57, 58]. The MO marker GALC/O1 and myelin components (e.g., MBP, MOG) were under-expressed in PM gliomas (Fig. 2A, Additional file 1: Fig. S7A). Further, the transcriptional regulators MYRF/C11ORF9 [59, 60] and SOX10 [61, 62], which are essential for the initiation and maintenance of myelination, were under-expressed in PM gliomas (Fig. 2A and Additional file 1: Fig. S7A).

In addition to the genes whose expression was previously shown to be associated with the PM glioma type [17, 18], additional genes that regulate the specification and maintenance of pre-OPC and OPC (ASCL1, CSPG4/NG2, DLL1, DLL3, HES6, PTPRZ1) and inhibitors of OPC differentiation or OL lineage terminal differentiation (ID4, SOX6 and GPR56) [42, 58, 63] were consistently enriched in PM gliomas as was the NFOL marker TCF7L2/TCF4 [64, 65] (Fig. 2B and C and Additional file 1: Fig. S7B and C). We also analyzed the expression profile for the makers of APC and mature astrocyte [21, 45–48] in PM gliomas. Enriched expression of APC markers (GLAST, FABP7 and FGFR3) and diminished expression of mature astrocyte markers (S100β, GLT1 and AQP4) were not observed in PM gliomas compared with NT brain tissues (Additional file 1: Fig. S8). Finally, co-staining with LFB (for myelin structure) and anti-IDH1 R132H antibody in 11 PM gliomas with 1p19q co-deletion and 9 PM gliomas without 1p19q co-deletion confirmed the absence of myelin fibers in tumor regions strongly stained with anti-IDH1 R132H antibody H09 (Fig. 2D and Additional file 1: Fig. S7D). As IDH-WT GBMs are committed in the NSC compartment [17], myelin structure was not detected in LFB staining of IDH-WT GBM samples (Additional file 1: Fig. S9).

These findings together show that PM gliomas, irrespective of their histological subtypes and malignant grades, and the composition of their genomic alterations, are blocked in the late stages of the oligodendrocyte lineage, and in particular have a defect in the myelination program.

### Differentiation blockage in PM gliomas analyzed at single cell level

**Fig. 3:**
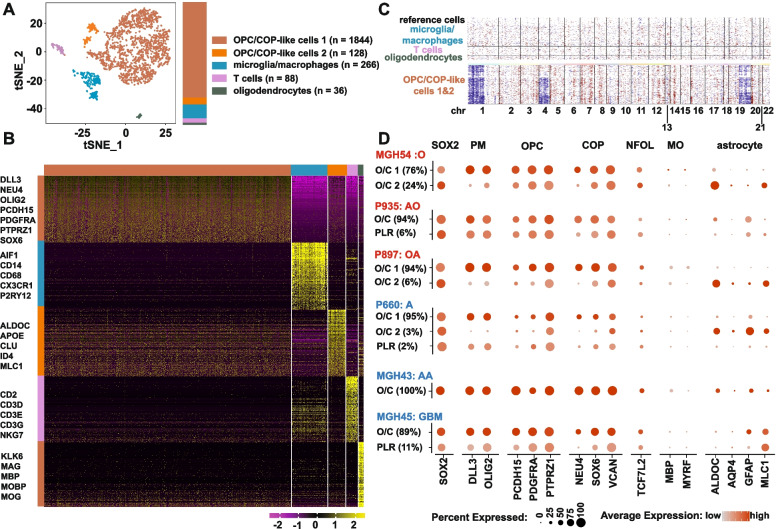
Uniform expression of OPC and COP markers in individual PM glioma cells. **A**–**C** Results of single-cell RNA-seq analysis of a representative PM glioma with 1p19q co-deletion from CGGA (sample ID: sc15). **A ***t*-SNE plot and cell numbers of the cell populations identified. **B** Heatmap of the top 200 most differentially expressed genes across the cell populations; lineage-specific hallmarks are shown. **C** Inferred chromosome CNVs in the OPC/COP-like cells with non-tumor cells as the control. **D** Concordant expression of SOX2 and early oligodendrocytic lineage markers but sporadic expression of astrocytic lineage markers in representative PM glioma samples with (MGH54, sc16 and sc15) or without 1p19q co-deletion (sc5, MGH43 and MGH45). O: oligodendroglioma grade 2, AO: anaplastic oligodendroglioma, OA: oligoastrocytoma grade 2, A:  astrocytoma grade 2, AA: anaplastic astrocytoma, O/C: OPC/COP-like cells, PLR: proliferating cells, APC: astrocyte precursor cells

To characterize the differentiation blockage at the single cell level, unsupervised clustering was performed using *t*-distributed stochastic neighbor embedding (*t*-SNE) [40] in single-cell RNA-seq data for 18 IDH*-*mutant gliomas and 8 IDH*-*wild-type gliomas from our own collection, complemented with 13 IDH*-*mutant gliomas [32, 33] and 7 IDH*-*wild-type gliomas [34] from previous reports (Fig. 3A, Additional file 1: Fig. S10A and Additional file 3: Table S2). Cell populations were defined according to differential expression of stage-specific markers of oligodendrocyte lineage and hall makers of microglia/macrophages and T cells (Fig. 3B and Additional file 1: Fig. S10B). Malignant cells were further recognized with SOX2 expression [44] and copy number variations (CNV) inferred from the average expression of genes in large chromosomal regions within each cell [32, 33] (Fig. 3C and D and Additional file 1: Fig. S10C and D). Though these glioma samples were diagnosed as different histological subtypes and grades, and single-cell RNA-seq data were generated using different platforms by different laboratories (Additional file 3: Table S2), malignant cell populations showed highly similar gene expression pattern under the EM/PM classification scheme. Markers of pre-OPC (DLL1, DLL3 and HES6), OPC (PCDH15, PDGFRA and PTPRZ1), and COP (NEU4, SOX6 and VCAN), but not markers of APC (FABP7, FGFR3 and GLAST), were nearly uniformly expressed in the malignant cells in comparison with non-malignant cells from all PM gliomas analyzed, irrespective whether they harbored 1p19q co-deletion or not (Fig. 3B and D and Additional file 1: Fig. S10B and D). In contrast, fewer malignant cells expressed the NFOL marker TCF7L2, and even fewer expressed the MO marker MYRF (Fig. 3D and Additional file 1: Fig. S10D). In a smaller population of malignant cells, astrocytic markers ALDOC, AQP4, GFAP, and MLC1 were sporadically expressed on a background of uniform expression of OPC and COP markers (the O/C2 population, Fig. 3B and D and Additional file 1: Fig. S10B, D).

Focusing only on the malignant populations, we also assessed the expression profile of the putative lineage signatures defined by Venteicher et al. [32]. Here, the signature of oligodendrocytic lineage (OC genes) was enriched in the population of OPC/COP-like cells 1 (O/C 1 cells), which constitute the major fraction of malignant cells, while the signature of astrocytic lineage (AC genes) was enriched in the minor population O/C 2 cells (Additional file 1: Fig. S11). As APC markers were not enriched in IDH-mutant glioma cells (Fig. 3D and Additional file 1: Fig. S10D), the expression of AC genes in O/C 2 cells would unlikely originate from APC, but more likely from the type-2 astrocyte differentiation potential of the OPC-like malignant cells [66, 67].

In 15 IDH*-*wild-type gliomas analyzed, malignant cells uniformly expressed SOX2 and harbored chromosome 7^gain^/chromosome 10^loss^ (Additional file 1: Fig. S12). In 6 IDH*-*wild-type gliomas, malignant cells showed up-regulated and concordant expression of astrocytic genes but sporadic expression of OPC and COP markers (Additional file 1: Fig. S12A-C and G). In the remaining 9 samples, an additional malignant cell population with concordant expression of early oligodendrocyte lineage markers but sporadic expression of astrocytic genes was observed (Additional file 1: Fig. S12D-G). In addition to sporadic and heterogeneous expression of OPC and COP markers, markers of NFOL (TCF7L2) and MOL (MBP, MYRF) are also sporadically and weakly expressed (Additional file 1: Fig. S12G).

Using canonical correlation analysis (CCA), we next compared the gene expression signatures of the PM glioma cells to the transcriptomes of normal cells in oligodendrocyte lineage differentiation [43]. In all 31 IDH-mutant gliomas analyzed, malignant cells exhibited strong similarity of expression patterns to OPC and/or COP, with Pearson correlation coefficient ranging from 0.63 to 0.99 (Additional file 4: Table S3).

**Fig. 4:**
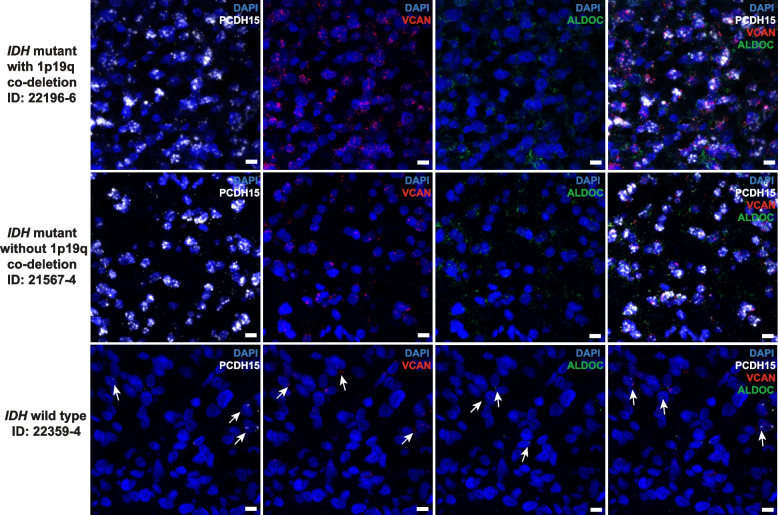
Expression of OPC and COP markers in individual PM glioma cells validated with RNAscope analysis. Cells from IDH-mutant/PM gliomas with or without 1p19q co-deletion uniformly co-expressed markers of OPC (PCDH15, white) and COP (VCAN, red); the expression of the astrocytic marker ALDOC (green) was sporadic. PCDH15 and VCAN were however sporadically expressed in IDH*-*wild-type/EM gliomas. RNAscope images of representative samples are shown, scale bar: 10 μm

RNAscope analysis in 6 PM and 4 EM samples confirmed that the vast majority of cells in PM gliomas co-expressed PCDH15 and VCAN. ALDOC was however expressed in a sporadic and infrequent pattern; very few cells in the EM gliomas expressed PCDH15 and VCAN (Fig. 4 and Additional file 1: Fig. S13).

These findings together reinforce the findings at the single cell level that PM glioma cells show enriched expression of pre-OPC, OPC, and COP signatures but are blocked at the premyelinating NFOL stage from further differentiation.

### Common differentiation stages in proliferating and non-proliferating PM glioma cells

**Fig. 5:**
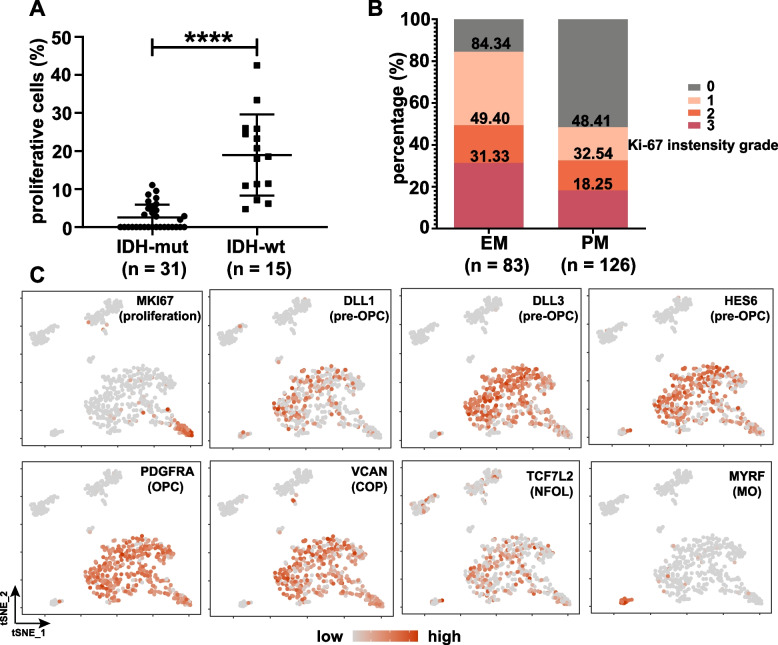
Common differentiation stages in proliferating and non-proliferating PM glioma cells.** A** Frequencies of proliferating cells in single-cell RNA-seq data from the PM and EM gliomas analyzed. **B** Ki-67 staining of the CGGA cohort. **C** Expression of cell proliferation or oligodendrocyte lineage stage-specific markers overlaid on the* t*-SNE map for one representative sample (MGH45)

Proliferating and non-proliferating cells in PM gliomas may reside at different differentiation stages. Focusing on the heterogeneities between the malignant cells, recent reports suggest a hierarchical model of IDH-mutant gliomas [32, 33], in which proliferating cells represent undifferentiated neural stem/progenitor cells and non-proliferating cells undergo differentiation along the astrocytic and oligodendrocytic lineages. We identified proliferating cells by concomitant expression of MKI67, TOP2A, CCNB2, and CDK1. However, cell proliferation was not detected in 17 of the 31 PM gliomas in our single-cell RNA-seq analyses. In the remaining 14 samples, 5.66 ± 2.75% of the malignant cells were proliferating. By contrast, each of the 15 EM gliomas analyzed contained proliferating cells, 18.96 ± 10.69% of the malignant cells were active in cell cycle (Fig. 5A and Additional file 3: Table S2). This is consistent with the Ki-67 staining results in a large CGGA cohort of clinical samples [27, 51], whereas Ki-67 staining was not detected in 48.4% of the PM gliomas (*N* = 126), 31% of the EM gliomas (*N* = 83) showed a high extent of Ki-67 staining (Fig. 5B and Additional file 1: Fig. S14), potentially accounting for the differential aggressiveness between the EM and PM gliomas [17, 18]. Notably, our analyses show that proliferating and non-proliferating PM glioma cells shared the same expression patterns for the markers of pre-OPCs, OPCs, COPs, and NFOLs, with a lack of expression in the markers of MO (Fig. 5C). These findings mirror our own observations (Additional file 1: Fig. S15) and other reports that only a small subset of OPCs and COPs in juvenile and adult mouse brain are in cell cycle [42, 43]. We also used principal component analysis (PCA) to assess the global transcriptomic difference between proliferating and non-proliferating cells. In this analysis, inclusion of non-malignant cells is essential for the characterization of the overall identity of malignant cells. Using non-malignant cells as the control, proliferating and non-proliferating cells did not form distinct populations but were overlapping to a larger extent (Additional file 1: Fig. S16). These findings indicate that proliferating and non-proliferating cells are highly similar at the global transcriptome level. Thus, proliferating and non-proliferating PM glioma cells share the same differentiation stages, and most of the PM glioma cells are in fact quiescent.

### Suppressed myelination program due to IDH mutation-induced DNA hypermethylation

**Fig. 6:**
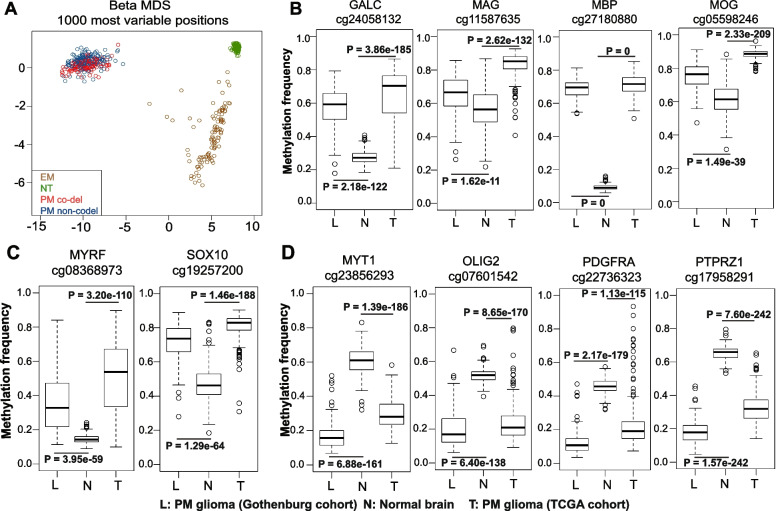
Suppressed myelination program in PM gliomas due to *IDH*-mutation induced DNA hypermethylation. **A** Multidimensional scaling (MDS) plot of the top 1000 most variable CpG sites in PM gliomas, EM gliomas, and normal brain tissues. **B** Hypermethylation of the MO marker GALC and the myelin components MAG, MBP, and MOG in PM gliomas. **C** Hypermethylation of MYRF/C11orf9 and SOX10 in PM gliomas. **D** Hypomethylation of OPC regulators MYT1, OLIG2, PDGFRA, and PTPRZ1 in PM gliomas. L, N, and T indicate PM glioma samples from Gothenburg, normal brain samples, and PM glioma samples from TCGA, respectively

**Fig. 7:**
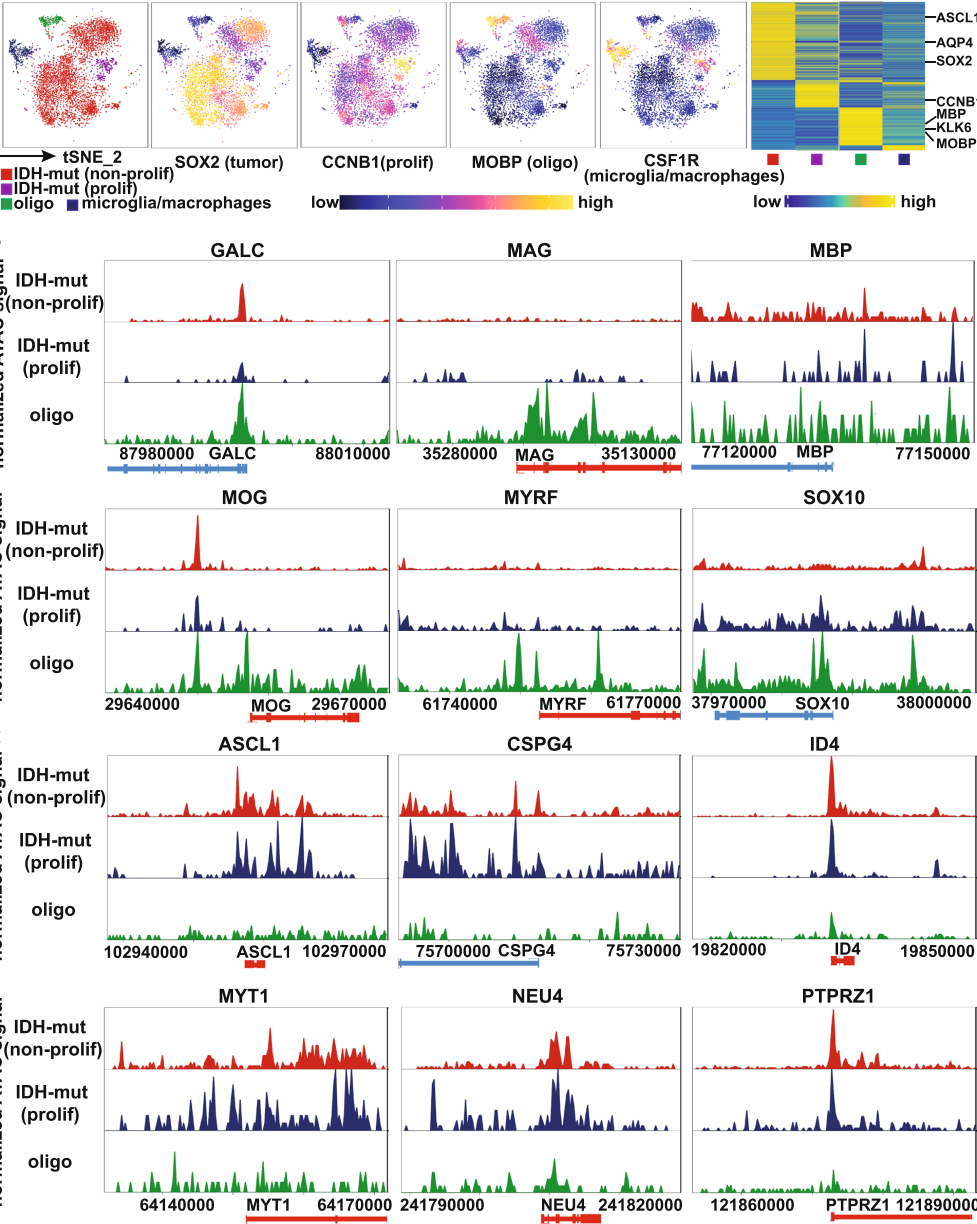
Chromatin status of key members of myelination program and OPC specification and maintenance in IDH-mutant gliomas. **A** Chromatin status-based identification of cell populations and the chromatin status of canonical markers across the respective cell populations identified. *t*-SNE plots and heatmap are shown. **B** Inaccessible chromatin status of oligodendrocyte marker, myelin components, and myelination-regulatory genes in both proliferating and non-proliferating IDH-mutant glioma cells. **C** Open chromatin status in genes involved in OPC specification and maintenance in both proliferating and non-proliferating IDH-mutant glioma cells. IDH-mut: IDH-mutant, non-prolif: non-proliferating cells, prolif: proliferating cells, oligo: oligodendrocytes

**Fig. 8:**
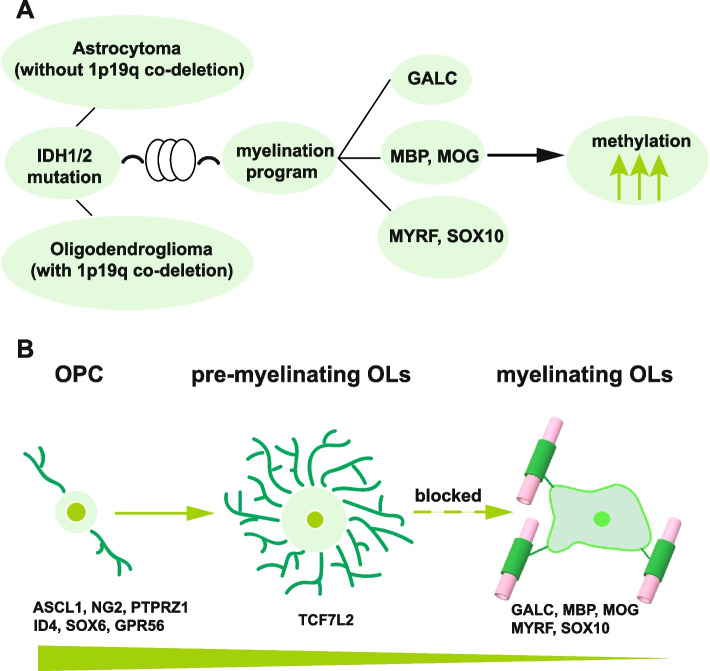
Schematic depiction of differentiation blockage of IDH-mutant gliomas. **A**, **B** IDH-mutant astrocytomas and oligodendrogliomas are both blocked at the premyelination stage due to hypermethylation and down-regulated expression of myelination regulators and myelin components. OPC: oligodendrocyte progenitor cell, OLs: oligodendrocytes

Gain-of-function mutations in *IDH1/2* are the only known genomic alterations shared between the two PM glioma subtypes and represent one of the founder events for all PM tumors [9, 68]. *IDH* mutations induce CpG island hypermethylation in acute myeloid leukemia (AML) [69], chondrosarcoma [70], and glioma [14, 71], resulting in blocked cell differentiation in AML [69] and chondrosarcoma [70]. Though previous studies suggest that hypermethylation in binding sites of insulator proteins may activate PDGFRA expression and thereby generate a proliferative advantage in IDH*-*mutant gliomas [72], the exact developmental pathways affected by *IDH* mutation-induced global DNA hypermethylation in gliomas are hitherto unclear. We compared the DNA methylomes from 280 PM and 103 EM gliomas from TCGA and 276 normal brain tissues from GSE43414 [53]. Consistent with previous findings [33], highly similar methylation patterns were found between PM gliomas with (*N* = 109) or without (*N* = 171) 1p19q co-deletion, with complete separation from EM gliomas and normal brain tissues (Fig. 6A). 53,869 differentially methylated positions (DMPs) with Benjamini–Hochberg adjusted *p* value < 0.05 and a methylation difference > 20% were identified between PM gliomas and normal brain tissues; 96% of these sites were hypermethylated in the PM gliomas, confirming the hypermethylator phenotype of IDH-mutant gliomas. These findings were validated in a local cohort containing 106 IDH-mutant gliomas from the Sahlgrenska University Hospital in Gothenburg, Sweden [52]. We also analyzed DNA methylation profile in IDH-wildtype GBM/EM samples. Among the top 70 genes harboring the most significant CpGs, 69 genes showed hypomethylation in IDH-wildtype GBM/EM samples compared with normal brain tissues (Additional file 1: Fig. S17). Thus, IDH-wild-type GBM/EM samples are associated with DNA hypomethylation. In IDH-mutant/PM gliomas, coordinated hypermethylation in the transcription start site (TSS)/CpG island regions was observed in MO marker (GALC/O1), myelin components (MAG, MBP, MOBP, MOG), and essential regulators of the myelination program (MYRF/C11orf9 [59, 60] and SOX10 [61]) (Fig. 6B and C, Additional file 1: Fig. S18 and Additional file 5: Table S4). In contrast, key regulators of OPC specification and maintenance [58], including MYT1, OLIG2, PDGFRA, PTPRZ1, SMOC1, and SOX8 were hypomethylated in PM gliomas (Fig. 6D, Additional file 1: Fig. S18 and Additional file 5: Table S4). These methylation profiles inversely mirrored the high expression of regulators of OPC specification and maintenance, and the under-expression of myelin components and regulators of myelination program. Further, we used single-cell ATAC-seq data from 9 IDH-mutant/PM gliomas reported by Babikir et al. [35] to analyze the chromatin status of the regulators of OPC specification and maintenance, as well as genes involved in myelination program. Based on the profiles of chromatin status, we identified 4 cell populations in these samples: proliferating IDH-mutant glioma cells (*n* = 438), non-proliferating IDH-mutant glioma cells (*n* = 4601), mature oligodendrocytes (*n* = 306), and microglia/macrophages (*n* = 464) (Fig. 7A). Comparing the chromatin status between proliferating or non-proliferating IDH-mutant glioma cells with mature oligodendrocytes, marker of mature oligodendrocytes (GALC), myelin components (MAG, MBP, and MOG), and myelination-regulating transcription factors (MYRF and SOX10) showed inaccessible chromatin (Fig. 7B), whereas regulators of OPC specification and maintenance (ASCL1, CSPG4, MYT1 and PTPRZ1), inhibitor of OPC differentiation or OL lineage terminal differentiation (ID4), and COP marker (NEU4) showed open chromatin (Fig. 7C). Though the results presented above should be further validated in experimental model, our findings suggest that *IDH* mutation-induced DNA hypermethylation causes a differentiation blockade in the late stage of oligodendrocyte lineage, whereas the specification and maintenance in early stages of oligodendrocyte lineage are largely unaffected (Fig. 8).

## Discussion

Mapping dysregulated brain developmental programs in glioma is crucial for understanding the basis of the pathogenesis involved and may help identify treatment options for glioma. We have used the known developmental program of glial lineages to identify the differentiation state of gliomas with IDH mutations. Such tumors account for ~ 50% of adult gliomas. Our findings show that irrespective of histological subtypes and grades, and the status of 1p19q co-deletion and other genomic alterations, cells of IDH-mutant gliomas uniformly express early lineage signatures spanning pre-OPC to NFOL and are stalled in oligodendrocyte differentiation due to suppressed myelination program, which is potentially caused by IDH mutation. Furthermore, cells of IDH-mutant gliomas are predominantly quiescent, and proliferating cells and non-proliferating cells share the same differentiation state.

These findings change our common conception of IDH-mutant gliomas from astrocytoma or oligodendroglioma to ontogeny-based PM subtype. With 1p/19q co-deletion as a landmark, IDH-mutant gliomas are currently diagnosed as astrocytoma or oligodendroglioma [4, 5]. However, histological subtypes and grades do not truly map gliomas to dysregulated neural lineages and differentiation stages, recent studies show that IDH-mutant astrocytomas and oligodendrogliomas share glial lineages and developmental hierarchies, though the exact developmental program(s) could not be ascertained [33]. Our findings show that gliomas with IDH mutation are committed to the oligodendrocyte lineage, with a common differentiation blockage at the post-mitotic premyelination stage. These gliomas are enriched in the signatures of pre-OPC, OPC, and COP, but show reduced expression of NFOL and MFOL signatures, and they lack expression of myelination regulators and myelin components; markers of APC and mature astrocyte are sporadically and randomly expressed on the background of co-expressed OPC and COP signatures. Differentiation blockage is most likely caused by IDH mutation-induced hypermethylation in essential regulators of myelination MYRF/C11orf9 [59] and SOX10 [61] and in myelin genes. As DNA methylation patterns show little intratumoral heterogeneity [73] and hypermethylation in myelination regulators and myelin genes are associated with inaccessible chromatin structure, blocked expression of myelination regulators and myelin genes in IDH-mutant gliomas appears to be causally repressed by IDH mutation.

Our findings are at odds with the widely discussed hierarchy model that malignant cells in IDH-mutant gliomas consist of three subpopulations: proliferative undifferentiatied stem/progenitor cells and nonproliferating cells differentiated along the astrocytic or oligodendrocytic lineages [32, 33]. This discrepancy most likely arises from the conceptual issues that the hierarchy model focused on the transcriptomic variations across the malignant cells or the tumor tissues without using non-malignant cells or tissues as the control [32, 33]. That approach is inefficient in capturing neural lineage and differentiation stage-related gene expression signatures, but rather capturing transcriptomic similarities and genomic alteration-related signatures [33]. Further, the transcriptomes of the malignant cells were scrutinized against a putative stemness signature and lineage-specific signature gene sets which are only partially consistent with differentiation programs as measured in mice [33]. In those lineage-specific signature gene sets, “genes associated with glial differentiation that do not correlate with the program in the tumor cells were removed, whereas other genes that are not known to be involved in glial differentiation but are coexpressed with the glial programs are added” [33]. In contrast, our findings are based on the transcriptomic analyses of IDH-mutant gliomas against a set of experimentally proven markers and fate determining regulators, and we made no alterations to the signature gene sets derived from purified glial cell populations.

In consistency with the reports of hierarchical model [32, 33], our analyses also identified a minor population of PM glioma cells with relatively enriched expression of astrocytic lineage genes proposed in the hierarchical model. However, markers of pre-OPC, OPC, and COP are also expressed in these cells. Further, genes concordantly enriched in both subtypes of PM gliomas do not show enriched expression in developing astrocyte. In the analyses of both bulk sample and single-cell transcriptomes, markers of APC are not enriched in PM gliomas. These findings together indicate that the expression of astrocytic lineage genes in a subset of PM glioma cells probably originate from the type 2 astrocyte differentiation potential of the early-stage oligodendrocyte precursors as the COO of these tumors [66, 67].

Our findings are expected to facilitate the studies of pathogenic mechanisms of IDH-mutant gliomas. The lineage identity and differentiation state of neural cells significantly impact the transformation capacity of glioma-associated tumor suppressors [74] and the biological behaviors of the respective tumor models [74–76]. Generating adequate models for IDH-mutant gliomas has so far proven to be challenging [77]. Our findings suggest that instead of introducing IDH mutations into astrocytes [71, 78–80] or neural stem/progenitor cells [81–83] with proliferative advantages as the main readout, introducing IDH mutations into the context of oligodendrocyte lineage differentiation could be more advantageous for understanding of the pathogenesis and vulnerabilities of IDH-mutant gliomas.

Finally, our findings are also expected to facilitate the development of subtype-specific treatment. Though IDH-mutant gliomas are currently stratified into prognostically relevant astrocytoma or oligodendroglioma [4], our findings show that both IDH-mutant astrocytomas and oligodendrogliomas are committed to the oligodendrocyte lineage and are blocked at the premyelination stage. They may thereby respond to the same treatment, as suggested by a recent report on the benefit from procarbazine, lomustine, and vincristine treatment in IDH-mutant oligodendrogliomas irrespective of 1p19q co-deletion status [84]. Further, as proliferative cells were not identified in > 50% of the IDH-mutant gliomas, anti-mitotic and DNA damaging adjuvant treatment may negatively impact the long-term outcome of these patients [85]. Instead, the blockade of the myelination program might serve as a vulnerable target in differentiation therapies against IDH-mutant gliomas.

## Conclusions

Taken together, we conclude that IDH-mutant astrocytomas and oligodendrogliomas are both committed to the oligodendrocyte lineage and are blocked at the premyelination stage due to hypermethylation and down-regulated expression of myelination regulators and myelin components. In these gliomas, the small fraction of proliferating malignant cells and the bulk malignant cells share a common differentiation stage. These findings may constitute a conceptual framework supporting future studies of pathogenesis and treatment development for gliomas and other cancers with these mutations [69, 70].

## Supplementary Information

**Additional file 1: Fig. S1.** Differential transcriptomic and mutational profiles in PM gliomas with or without 1p19q co-deletion. **Fig. S2.** Declining expression of NFOL and MFOL signatures in PM gliomas from GSE4290.** Fig. S3.** Declining expression of NFOL and MFOL signatures in PM gliomas from the REMBRANDT dataset. **Fig. S4.** Genes upregulated in the PM gliomas were enriched in murine OPCs. **Fig. S5.** Genes concordantly enriched in both PM subtypes were differentially expressed between OPC and MO, but not between developing astrocyte and mature astrocyte. **Fig. S6.** Enrichment of gene ontology terms in genes concordantly upregulated in the PM gliomas as shown in Fig. 1C. **Fig. S7.** Suppressed myelination program in PM gliomas from GSE4290. **Fig. S8. ** No distinct expression of astrocytic lineage genes between PM gliomas and NT brain tissues. **Fig. S9.** Co-staining of LFB and hematoxylin-eosin of representative IDH-WT GBMs.** Fig. S10. ** Uniform expression of early oligodendrocytic lineage markers in individual PM glioma cells from additional samples. **Fig. S11.** Enriched expression of previously reported oligodendrocytic (OC) lineage signature and astrocytic (AC) lineage signature in O/C1 and O/C2 cell populations, respectively. **Fig. S12.** Sporadic or heterogeneous expression of OPC and COP markers in individual EM glioma cells. **Fig. S13.** Expression of OPC and COP markers in individual PM glioma cells validated with RNAscope analysis. **Fig. S14.** EM and PM gliomas show marked difference in cell proliferation as assessed with Ki-67 staining in clinical samples. **Fig. S15.** OPCs and COPs in juvenile and adult brain are sporadically in cell cycle. **Fig. S16.** Proliferating and non-proliferating IDH-mutant glioma cells are similar at the global transcriptome level. **Fig. S17.** Top 70 genes with most significant CpGs in IDH-wildtype GBMs. **Fig. S18.** Myelination profiles of myelination regulator, hallmarks of MO and regulators of OPC specification and maintenance in PM gliomas.

**Additional file 2: Table S1. **Morphological subtypes found in the PM gliomas.

**Additional file 3: Table S2. **Frequencies of cell populations in single cell RNA-seq samples analyzed.

**Additional file 4: Table S3. **Pearson correlation coefficient between PM glioma cells and cell populations in murine oligodendrocyte lineage.

**Additional file 5: Table S4. **Hypermethylation in myelination program but hypomethylation in regulators of OPC specification in PM gliomas.

## Data Availability

The bulk transcriptome data from the Chinese Glioma Genome Atlas (CGGA) were described previously [27, 86, 87] and deposited under GSE48865 (https://www.ncbi.nlm.nih.gov/geo/query/acc.cgi?acc=GSE48865). STRT-seq protocol-based single-cell RNA-seq data of glioma samples were recently reported and deposited under GSE117891 (https://www.ncbi.nlm.nih.gov/geo/query/acc.cgi?acc=GSE117891) [88] and can also be downloaded from CGGA website (http://cgga.org.cn/download.jsp). 10X protocol-based single-cell RNA-seq data of glioma samples supporting the current study are deposited under GSE227718 (https://www.ncbi.nlm.nih.gov/geo/query/acc.cgi?acc=GSE227718) [89]. The DNA methylation data are deposited under GSE175877 (https://www.ncbi.nlm.nih.gov/geo/query/acc.cgi?acc=GSE175877) [52]. All the other data and its information files supporting the findings of this study are available within the article and from the corresponding author upon reasonable request.
