# Supplementary material for: Stalled oligodendrocyte differentiation in IDH-mutant gliomas
Source: Genome Med. 2023 Apr 13;15:24. doi: 10.1186/s13073-023-01175-6 (PMC10103394; doi:10.1186/s13073-023-01175-6)
Supplement: Supplementary file 1 — Additional file 1: Fig. S1. Differential transcriptomic and mutational profiles in PM gliomas with or without 1p19q co-deletion. Fig. S2. Declining expression of NFOL and MFOL signatures in PM gliomas from GSE4290. Fig. S3. Declining expression of NFOL and MFOL signatures in PM gliomas from the REMBRANDT dataset. Fig. S4. Genes upregulated in the PM gliomas were enriched in murine OPCs. Fig. S5. Genes concordantly enriched in both PM subtypes were differentially expressed between OPC and MO, but not between developing astrocyte and mature astrocyte. Fig. S6. Enrichment of gene ontology terms in genes concordantly upregulated in the PM gliomas as shown in Fig. 1C. Fig. S7. Suppressed myelination program in PM gliomas from GSE4290. Fig. S8. No distinct expression of astrocytic lineage genes between PM gliomas and NT brain tissues. Fig. S9. Co-staining of LFB and hematoxylin-eosin of representative IDH-WT GBMs. Fig. S10. Uniform expression of early oligodendrocytic lineage markers in individual PM glioma cells from additional samples. Fig. S11. Enriched expression of previously reported oligodendrocytic (OC) lineage signature and astrocytic (AC) lineage signature in O/C1 and O/C2 cell populations, respectively. Fig. S12. Sporadic or heterogeneous expression of OPC and COP markers in individual EM glioma cells. Fig. S13. Expression of OPC and COP markers in individual PM glioma cells validated with RNAscope analysis. Fig. S14. EM and PM gliomas show marked difference in cell proliferation as assessed with Ki-67 staining in clinical samples. Fig. S15. OPCs and COPs in juvenile and adult brain are sporadically in cell cycle. Fig. S16. Proliferating and non-proliferating IDH-mutant glioma cells are similar at the global transcriptome level. Fig. S17. Top 70 genes with most significant CpGs in IDH-wildtype GBMs. Fig. S18. Myelination profiles of myelination regulator, hallmarks of MO and regulators of OPC specification and maintenance in PM gliomas. [file 13073_2023_1175_MOESM1_ESM.docx]

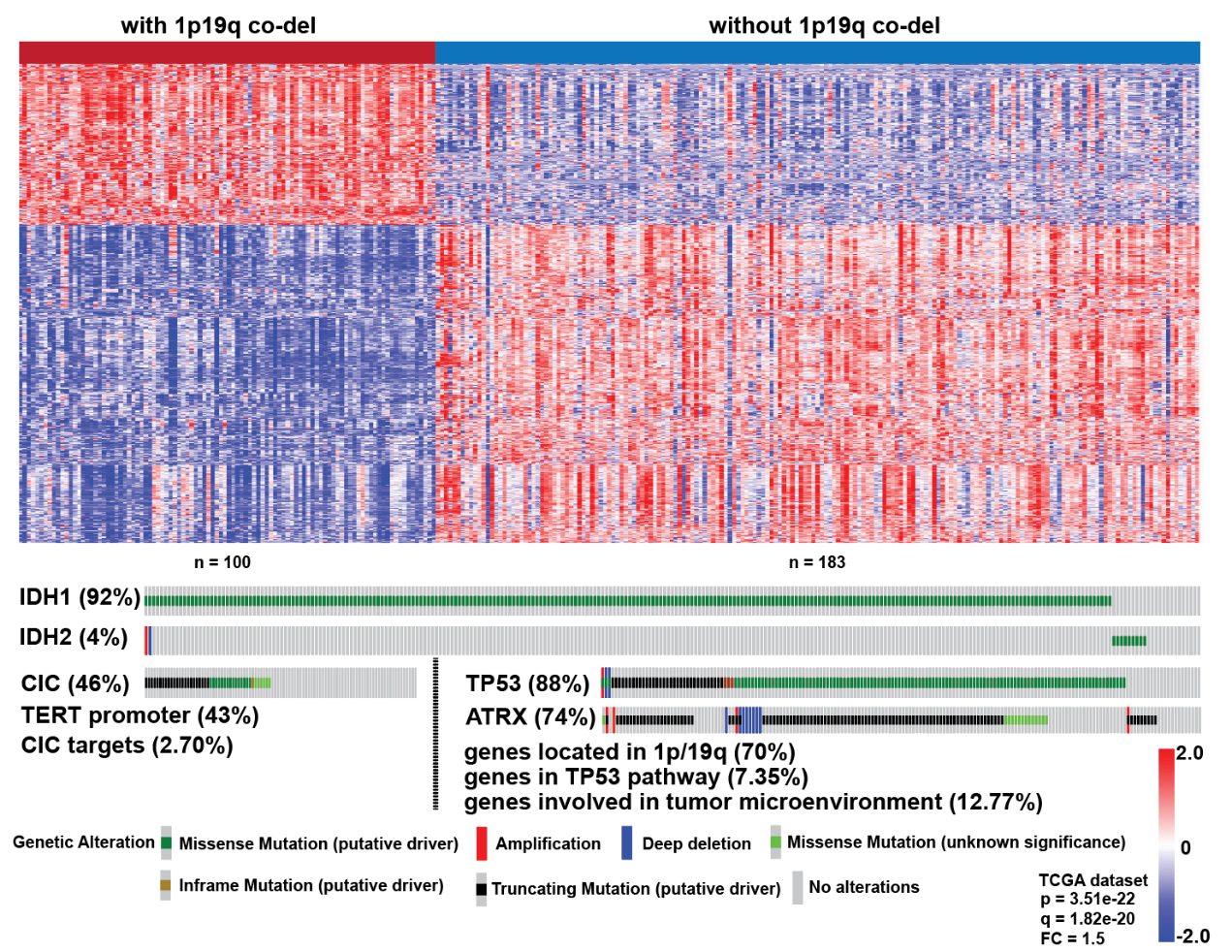
**Fig. S1.** **Differential transcriptomic and mutational profiles in PM gliomas with or without 1p19q co-deletion**.

Transcriptomes of 702 gliomas from TCGA were classified according to the EM/PM classification scheme. PM gliomas with 1p19q co-deletion (N = 100) or without 1p19q co-deletion (N = 183) were compared for their transcriptomic differences (upper) and profiles in canonical mutations (lower). PM gliomas with 1p19q co-deletion were enriched in targets of *CIC* mutation (e.g., ETV4 and ETV5); whereas PM gliomas without 1p19q co-deletion were enriched in genes located in 1p and 19q, members of TP53 pathway, and tumor microenvironment activities.


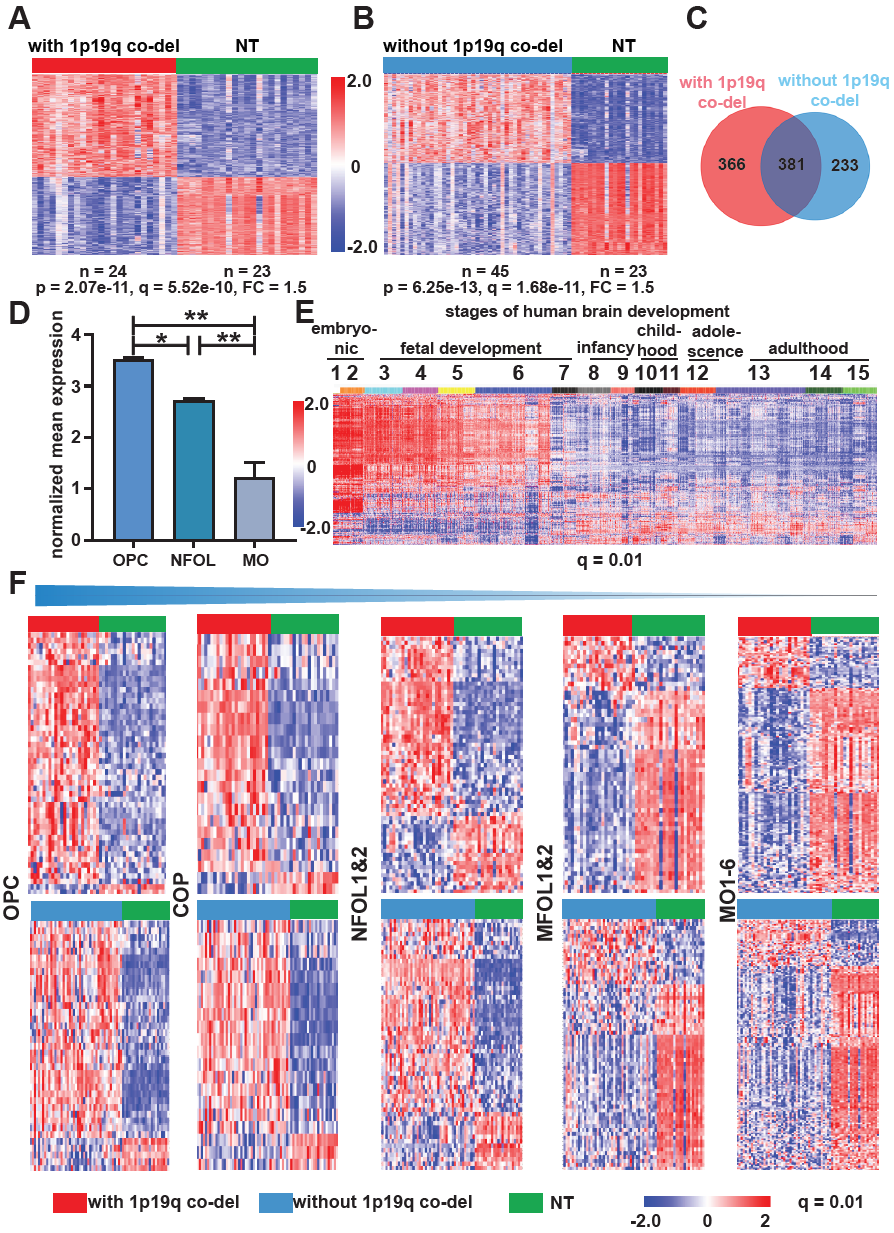


**Fig. S2. Declining expression of NFOL and MFOL signatures in PM gliomas from GSE4290.**

(**A, B**) Heatmaps of differential gene expression profile between PM gliomas and NT brain tissues at the indicated p and q values with a fold change (FC) at 1.5. Genes concordantly enriched in both PM glioma subtypes (**C**) show upregulated expression in OPC and NFOL of mouse oligodendrocyte lineage as analyzed in GSE52564 [1] (**D**), and in early stages of human brain development as analyzed in GSE25219 [2] (**E**). (**F**) Drastically declining expression in the gene expression signatures of NFOL and MFOL in PM gliomas with (upper) or without (lower) 1p19q co-deletion. The same color codes were used in panels A, B, C and F.


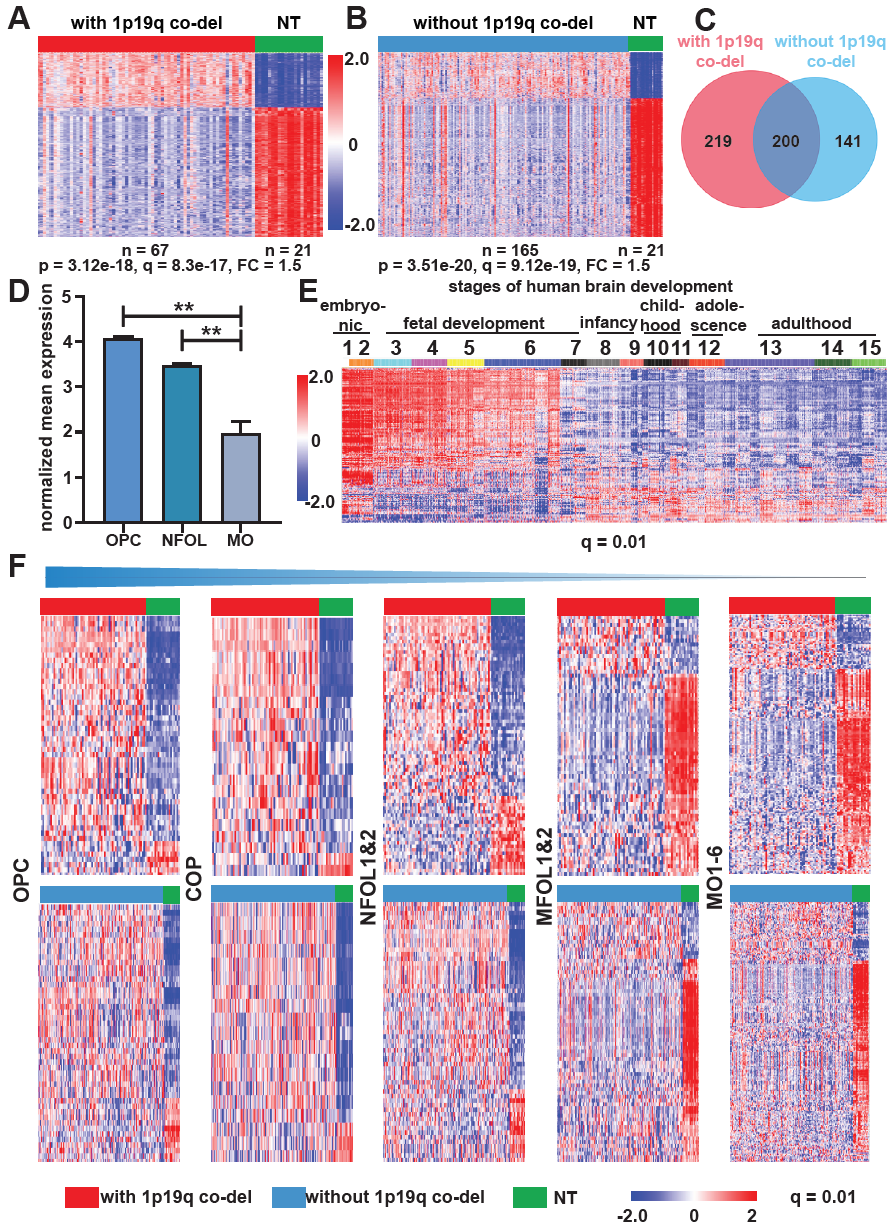


**Fig. S3. Declining expression of NFOL and MFOL signatures in PM gliomas from the REMBRANDT dataset.**

(**A, B**) Heatmaps of differential gene expression profile between PM gliomas and NT brain tissues at the indicated p and q values with a fold change (FC) at 1.5. Genes concordantly enriched in both PM glioma subtypes (**C**) show upregulated expression in OPC and NFOL of mouse oligodendrocyte lineage as analyzed in GSE52564 [1] (**D**), and in early stages of human brain development as analyzed in GSE25219 [2] (**E**). (**F**) Drastically declining expression in the gene expression signatures of NFOL and MFOL in PM gliomas with (upper) or without (lower) 1p19q co-deletion. The same color codes were used in panels A, B, C and F.


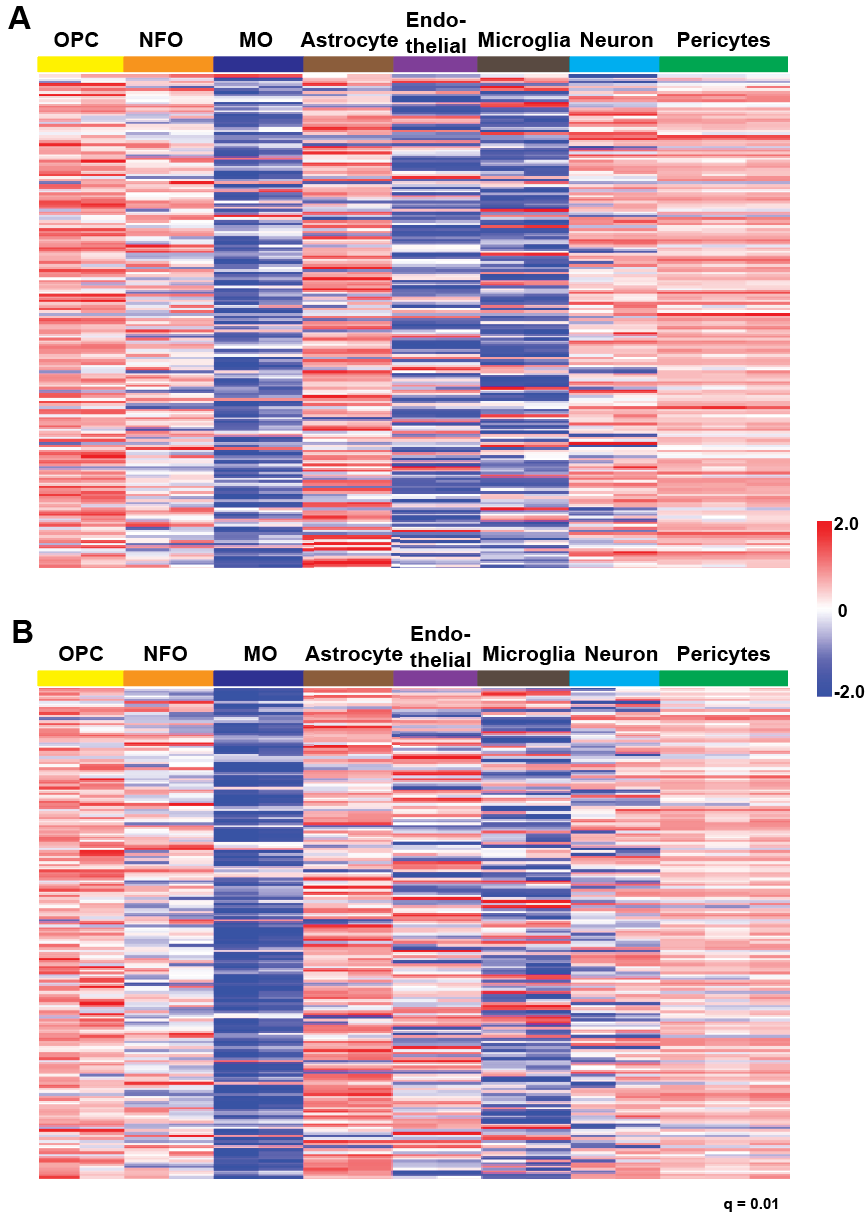
**Fig. S4.** **Genes upregulated in the PM gliomas were enriched in murine OPCs.** Expression profile of genes upregulated in PM gliomas with 1p19q co-deletion (upper) or without 1p19q co-deletion (lower) as identified in Fig. 1A and 1B, respectively, in murine neural cell types as analyzed in GSE52564 [1]. As pericytes may also originate from PDGFRA-positive neural progenitor cells [3], genes upregulated in PM gliomas were enriched in OPCs and also in pericytes, q = 0.01.


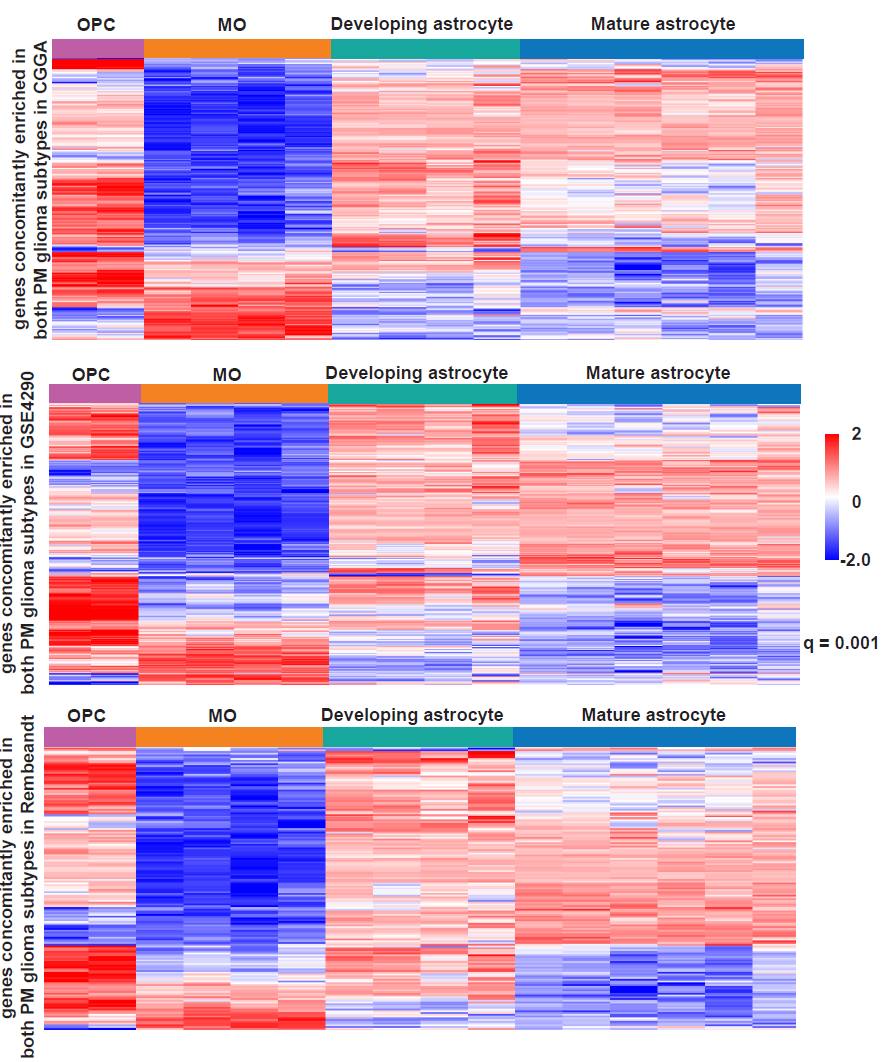
**Fig. S5. Genes concordantly enriched in both PM subtypes were differentially expressed between OPC and MO, but not between developing astrocyte and mature astrocyte.**

Heatmaps of the expression patterns of the genes concordantly enriched in both PM subtypes across the indicated neural cell populations at q = 0.001 are shown. OPC: oligodendrocyte progenitor cell; MO: mature oligodendrocyte. Data are derived from analyses using GSE9566 [4].


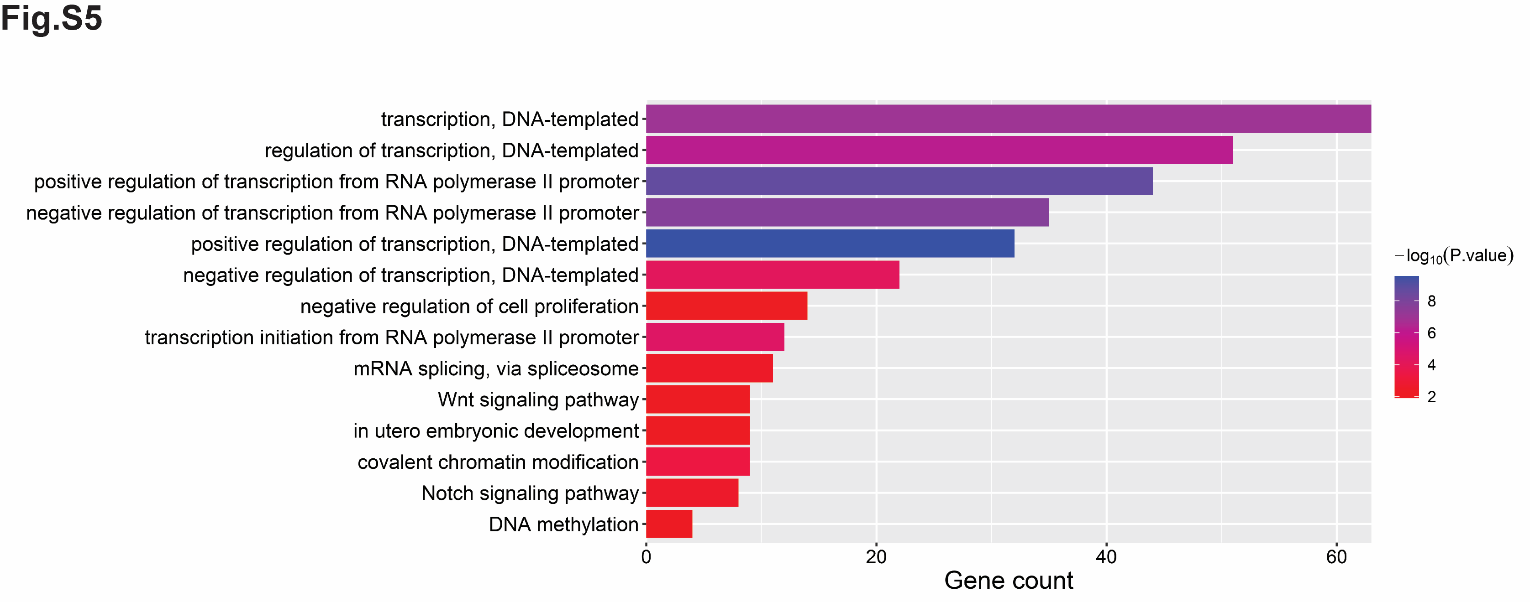
**Fig. S6**. **Enrichment of gene ontology terms in genes concordantly upregulated in the PM gliomas as shown in Fig. 1C.**

**
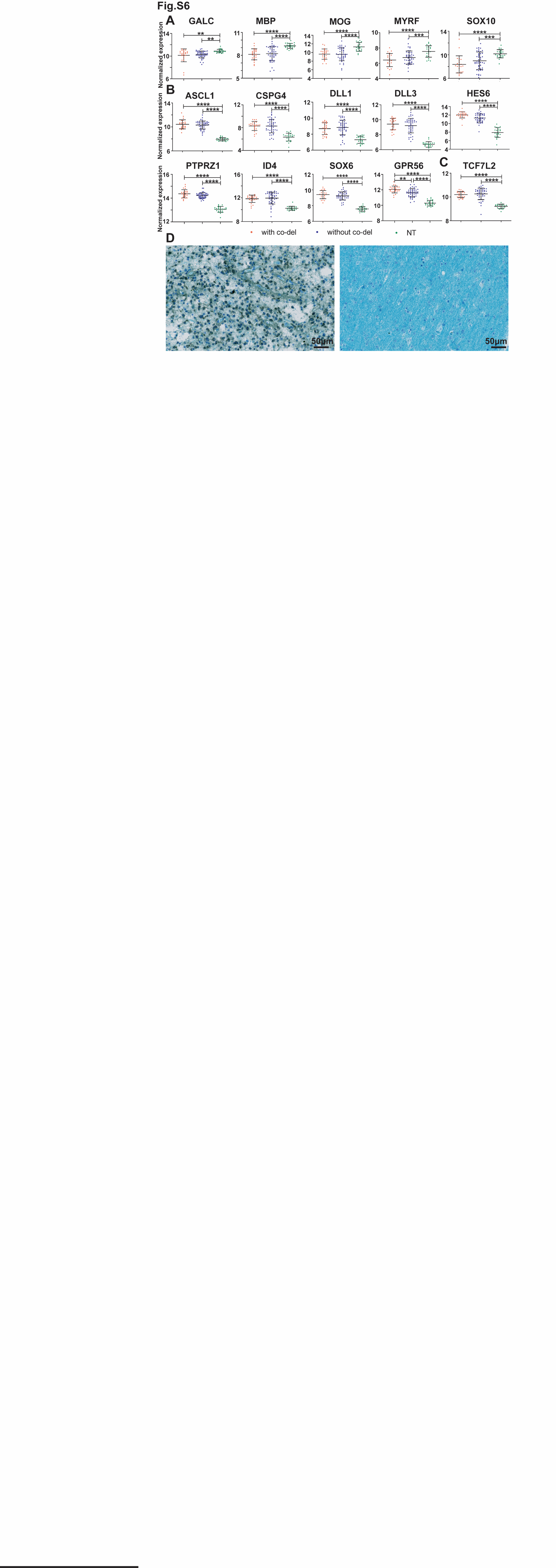
Fig. S7. Suppressed myelination program in PM gliomas from GSE4290.**

(**A**) Under expression of the MO marker GALC/O1, myelin components (MBP, MOG) and myelination regulators MYRF/C11ORF9 and SOX10 in PM gliomas. (**B**) Sustained expression of stage-specific markers of Pre-OPC and OPC in PM gliomas. (**C**) Sustained expression of stage-specific marker of NFOL in PM gliomas. (**D**) Co-staining with LFB which labels myelin structures, and with the anti-IDH1 R132H antibody which stains IDH1 mutant cells, of a representative PM glioma with 1p19q co-deletion is shown for tumor (left) and NT (right) regions. Myelin fiber is absent in tumor regions with strong cytosolic staining of the IDH1 R132H mutant protein.


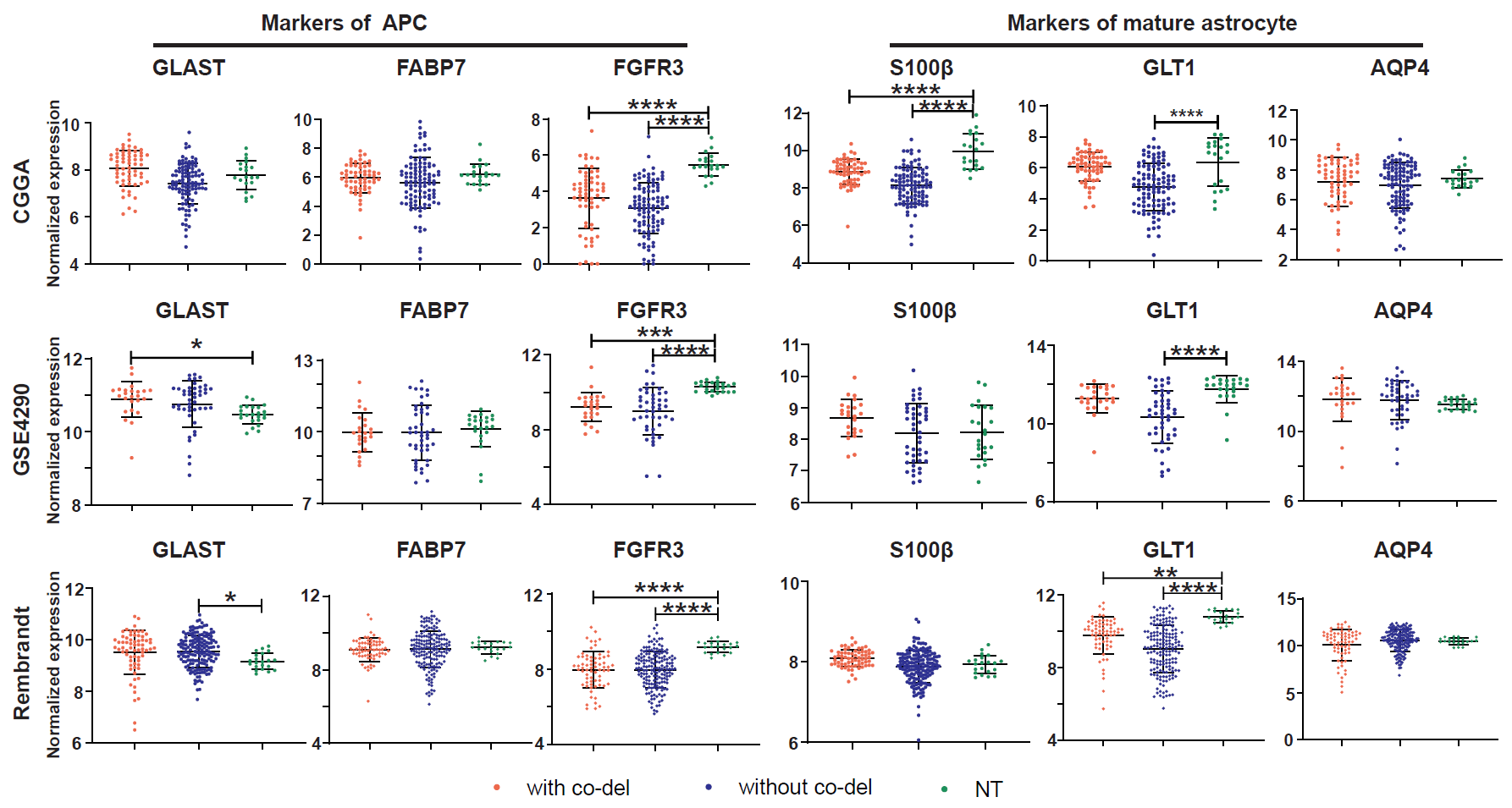
**Fig. S8. No distinct expression of astrocytic lineage genes between PM gliomas and NT brain tissues**.

Expression profiles of APC markers (GLAST, FABP7 and FGFR3) and mature astrocyte markers (S100β, GLT1 and AQP4) were analyzed between PM gliomas and NT brain tissues. With co-del: with 1p19q co-deletion; without co-del: without 1p19q co-deletion; NT: non tumor. *: p<0.05; **: p<0.01; ***: p<0.001; ****: p<0.0001.

**
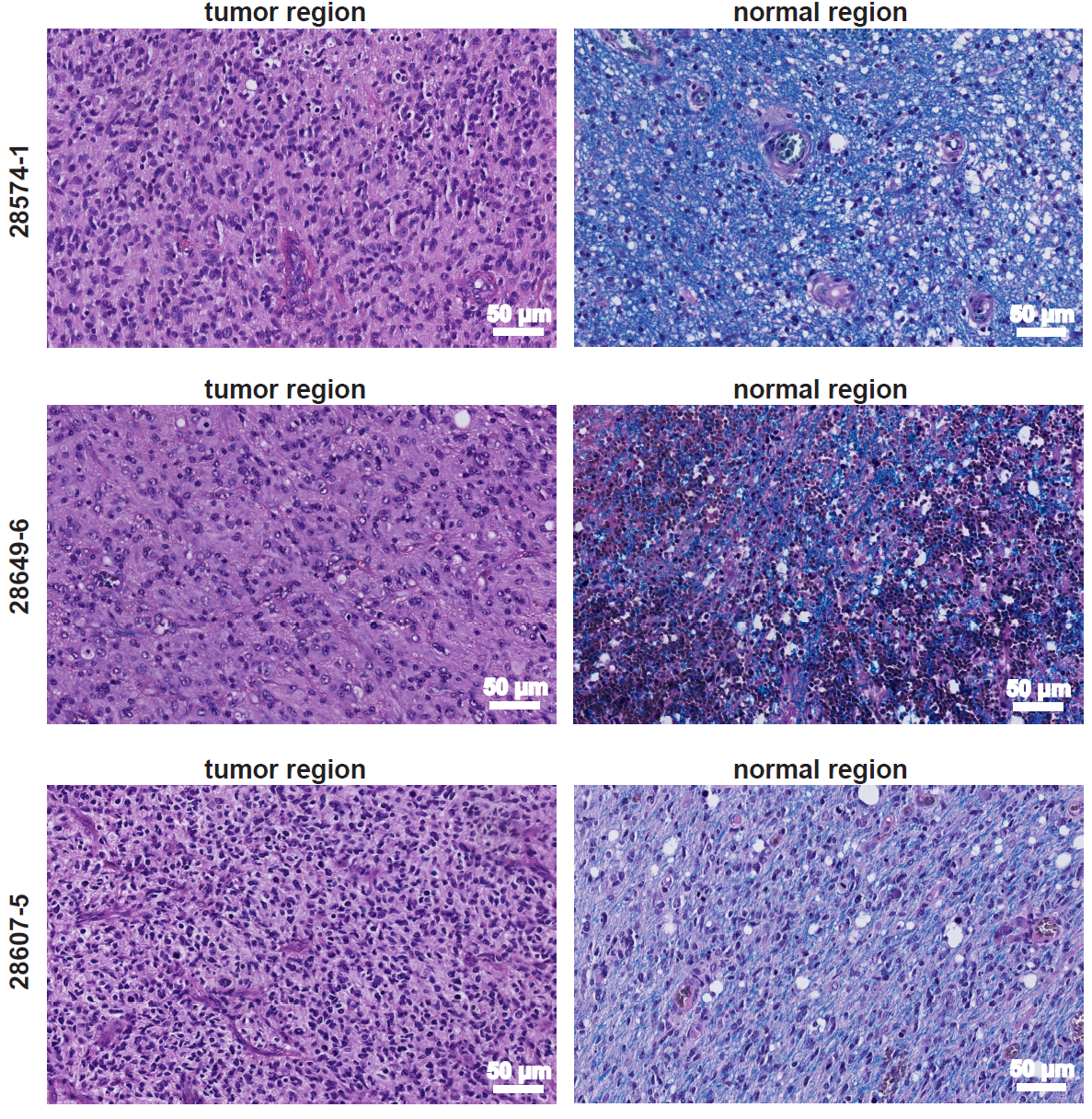
Fig. S9. Co-staining of LFB and hematoxylin-eosin of representative IDH-WT GBMs.**

LFB labelled myelin structures are absent in tumor regions.


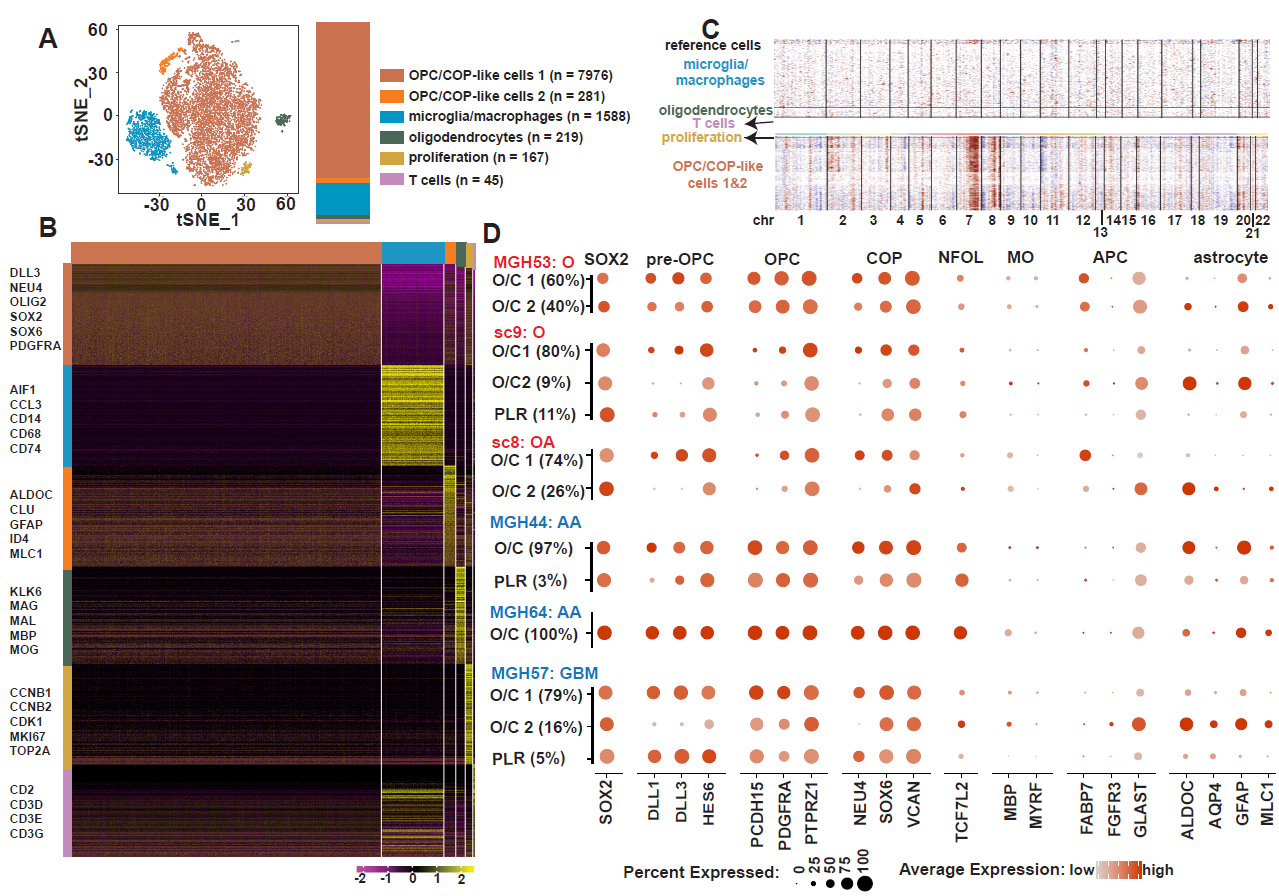


**Fig. S10. Uniform expression of early oligodendrocytic lineage markers in individual PM glioma cells from additional samples.**

(**A**) to **(C)** Results of single cell RNA-seq analysis of a representative PM glioma without 1p19q co-deletion from CGGA (sample ID: sc5). (**A**) *t*-SNE plot and cell numbers of the cell populations identified. (**B**) heatmap of the top 200 most differentially expressed genes across the cell populations, lineage-specific hallmarks are shown. **(C)** Inferred chromosome CNVs in the OPC/COP-like cells with non-tumor cells as the control. **(D)** Concordant expression of SOX2 and early oligodendrocytic lineage markers but sporadic expression of APC or mature astrocyte markers in additional representative PM glioma samples with (MGH53, sc9 and sc8) or without 1p19q co-deletion (MGH44, MGH64 and MGH57). O: oligodendroglioma grade 2, AO: anaplastic oligodendroglioma, OA: oligoastrocytoma grade 2, A: astrocytoma grade 2, AA: anaplastic astrocytoma. O/C: OPC/COP-like cells, PLR: proliferating cells. APC: astrocyte precursor cells.


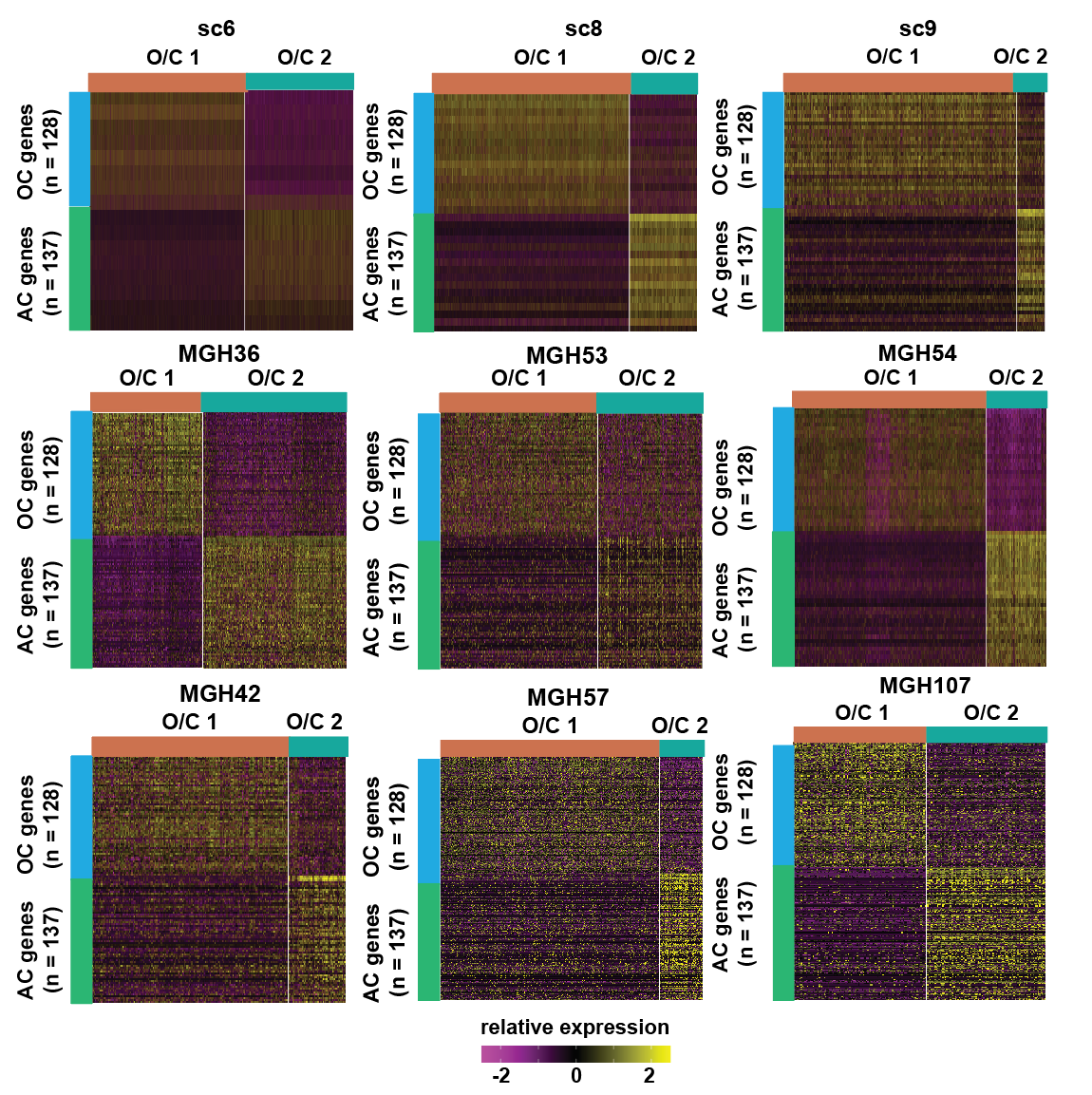
**Fig. S11. Enriched expression of previously reported oligodendrocytic (OC) lineage signature and astrocytic (AC) lineage signature in O/C1 and O/C2 cell populations, respectively**. Heatmaps of indicated IDH-mutant glioma samples are shown, O/C: OPC/COP-like.


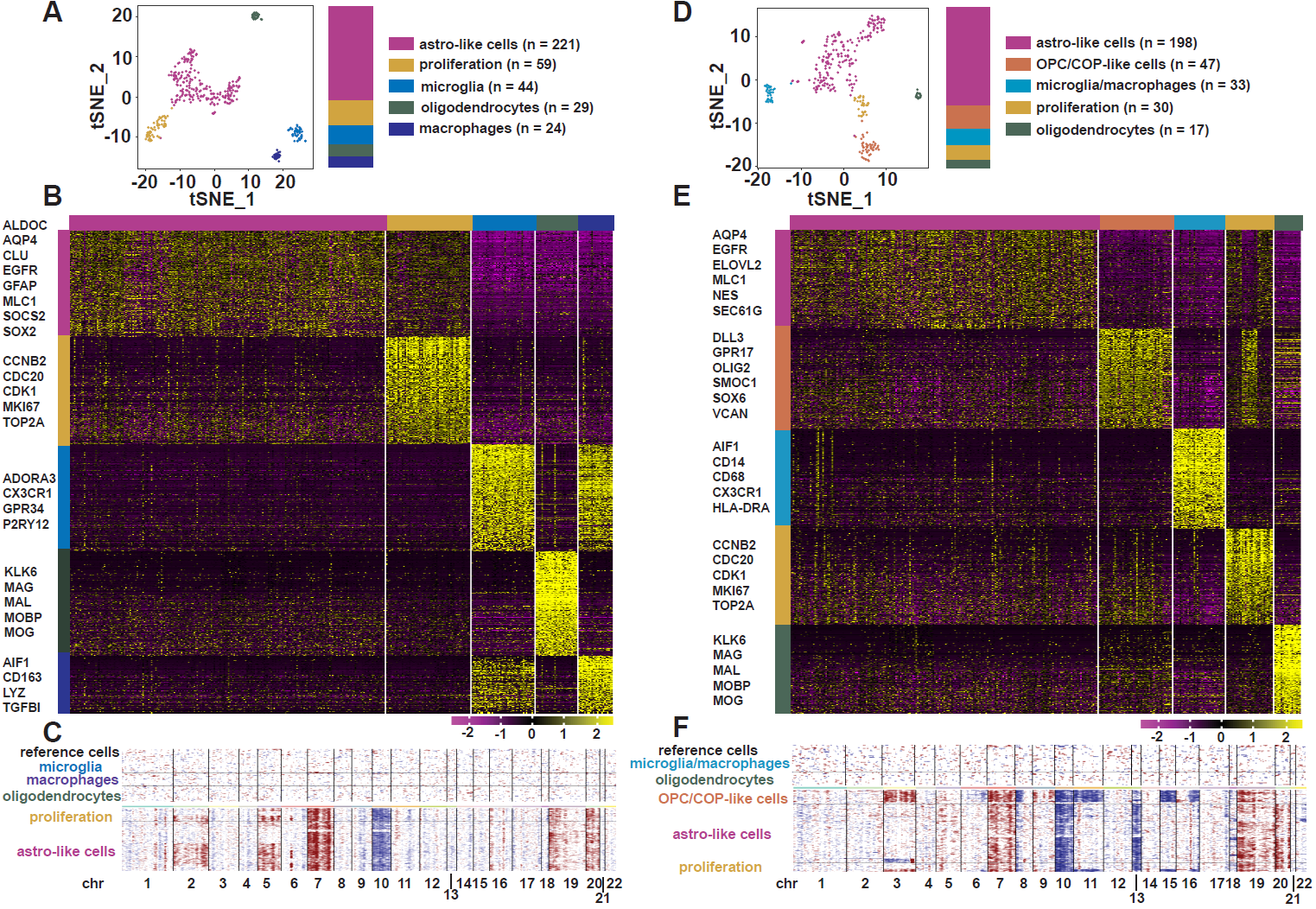


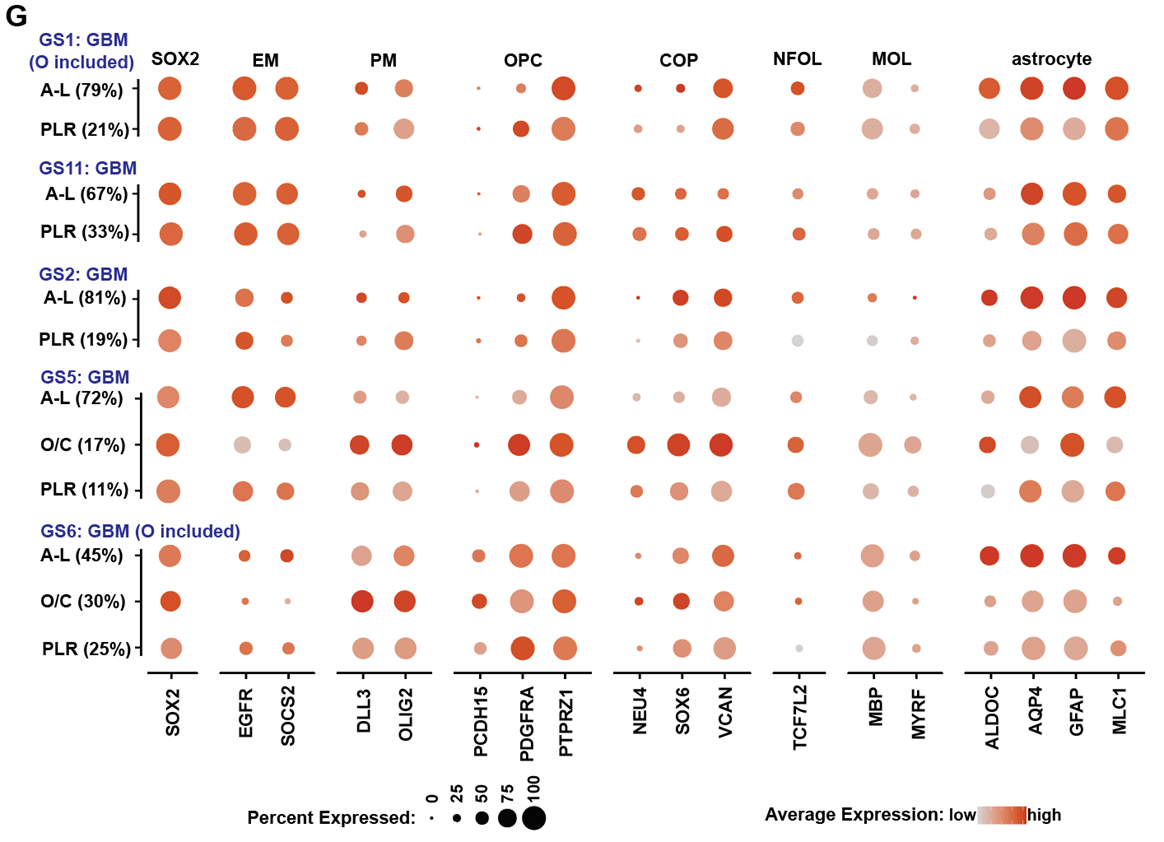
**Fig. S12. Sporadic or heterogeneous expression of OPC and COP markers in individual EM glioma cells.**

(**A**) to **(C)** Results of single cell RNA-seq analysis of a representative EM glioma with astro-like cells as the malignant cell population (sample ID: GS1). (**A**) *t*-SNE plot and cell numbers of the cell populations identified. (**B**) heatmap of the top 200 most differentially expressed genes across the cell populations, lineage-specific hallmarks are shown. **(C)** Inferred chromosome CNVs in the astro-like cells and proliferative cells with non-tumor cells as the control. (**D**) to **(F)** Results of single cell RNA-seq analysis of a representative EM glioma with both astro-like cells and OPC/COP-like cells as the malignant cell population (sample ID: GS5). (**D**) *t*-SNE plot and cell numbers of the cell populations identified. (**E**) heatmap of the top 200 most differentially expressed genes across the cell populations, lineage-specific hallmarks are shown. **(F)** Inferred chromosome CNVs in the astro-like cells, OPC/COP-like cells, and proliferative cells with non-tumor cells as the control. **(G)** Frequency and extent of the expression of SOX2, EGFR, SOCS2, stage-specific markers of oligodendrocyte lineage and astrocytic markers in malignant cells from indicated samples. A-L: astro-like cells, PLR: proliferating cells, O/C: OPC/COP-like cells.


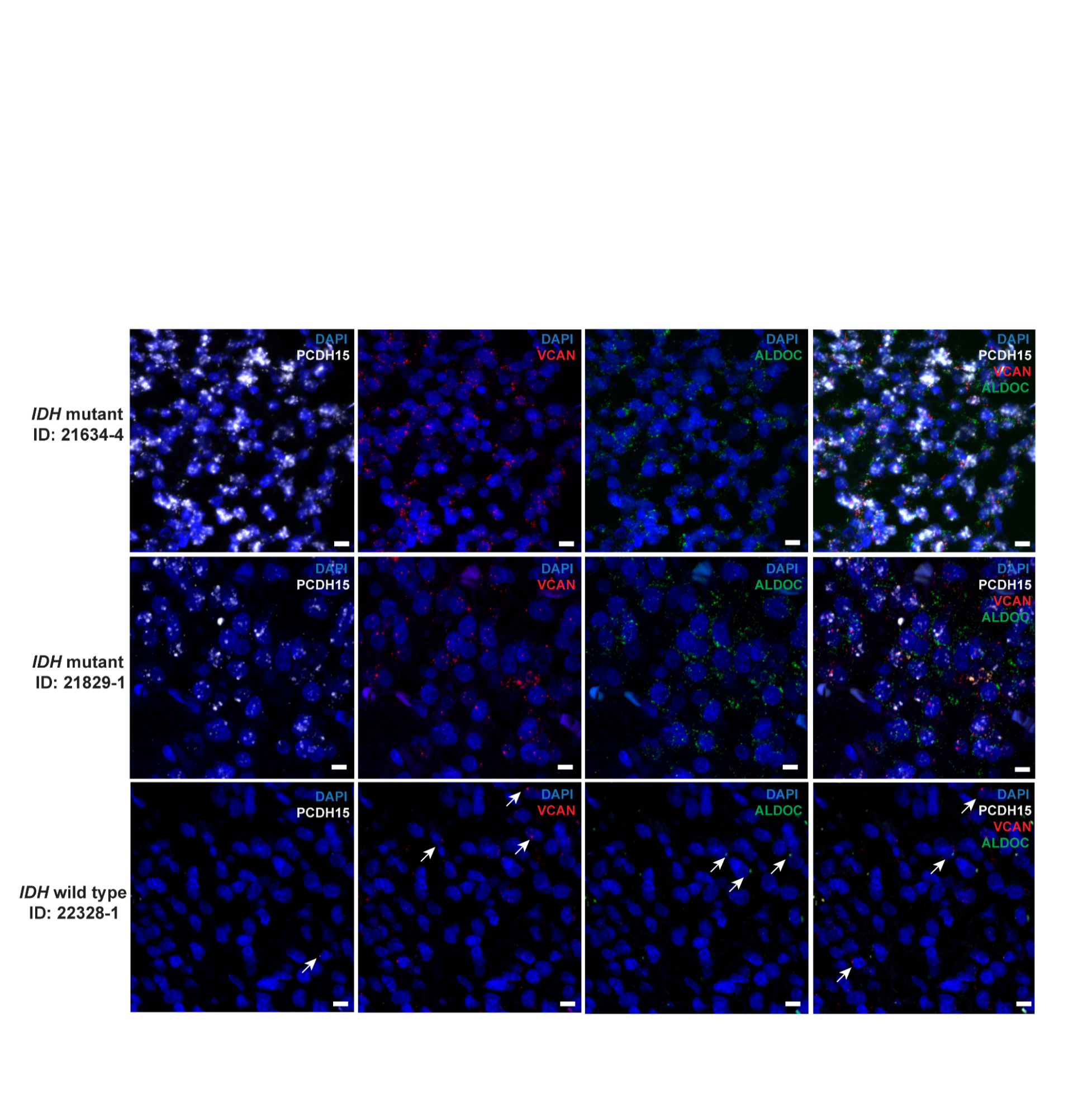


**Fig. S13. Expression of OPC and COP markers in individual PM glioma cells validated with RNAscope analysis.**

Cells from IDH*-*mutant/PM gliomas with or without 1p19q co-deletion uniformly co-expressed markers of OPC (PCDH15, white) and COP (VCAN, red), the expression of the astrocytic marker ALDOC (green) was sporadic. PCDH15 and VCAN were however sporadically expressed in IDH-wild type/EM gliomas. Data shown are RNAscope images of additional samples analyzed as shown in Fig. 4, scale bar: 10 μm.

**
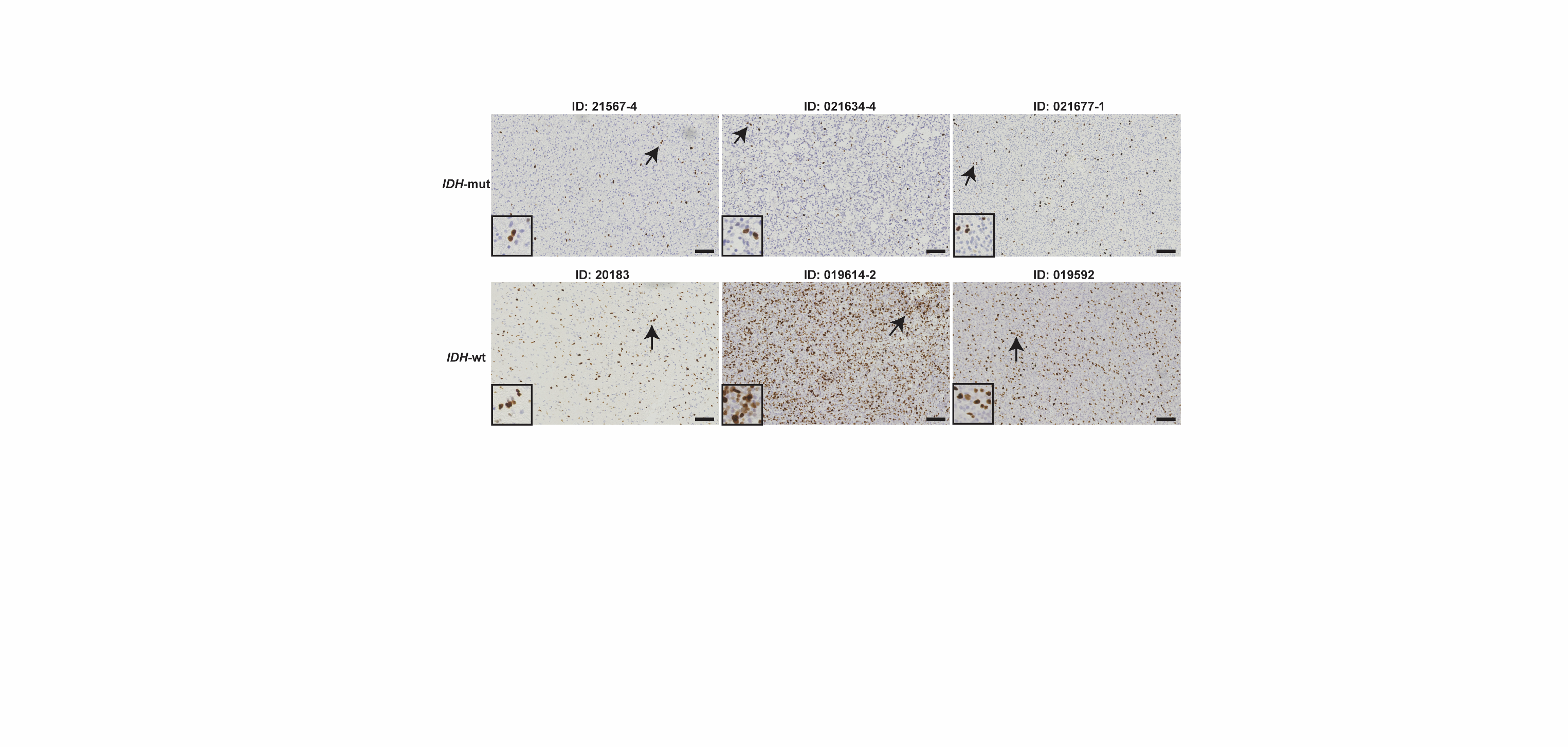
**

**Fig. S14. EM and PM gliomas show marked difference in cell proliferation as assessed with Ki-67 staining in clinical samples.**

Images of Ki-67 staining from representative IDH-mutant/PM gliomas (upper panel) or IDH-wild type/EM gliomas (lower panel) are shown.

**
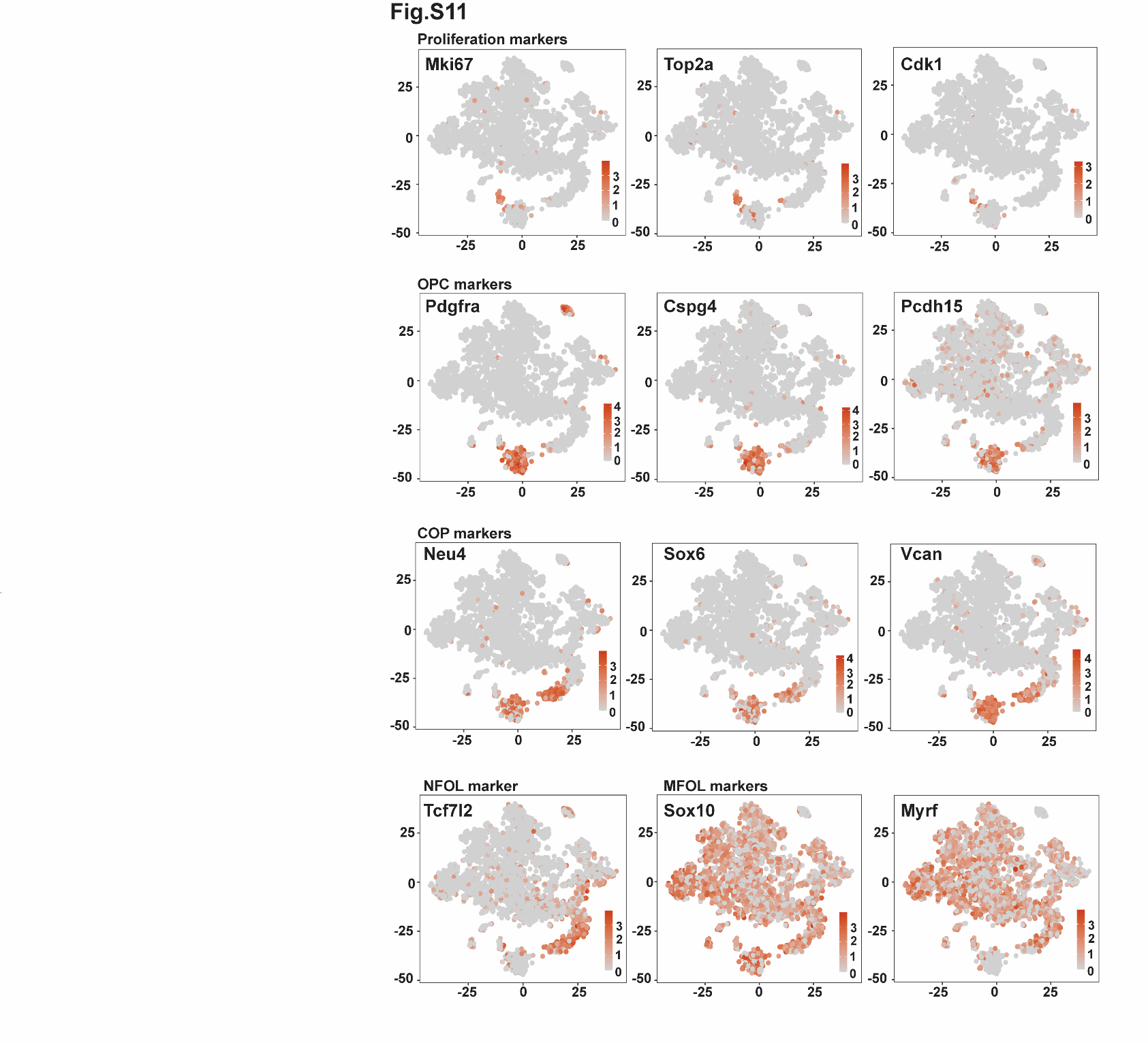
Fig. S15. OPCs and COPs in juvenile and adult brain are sporadically in cell cycle.** Single cell RNA-seq data from oligodendrocyte lineage cells from juvenile and adult mouse, GSE75330 [5] were analyzed for the expression pattern of cell proliferation markers and§§ stage-specific markers of oligodendrocyte lineage. Marker expression overlaid on the t-SNE map of cell populations is shown. Very few OPCs and COPs are proliferating.


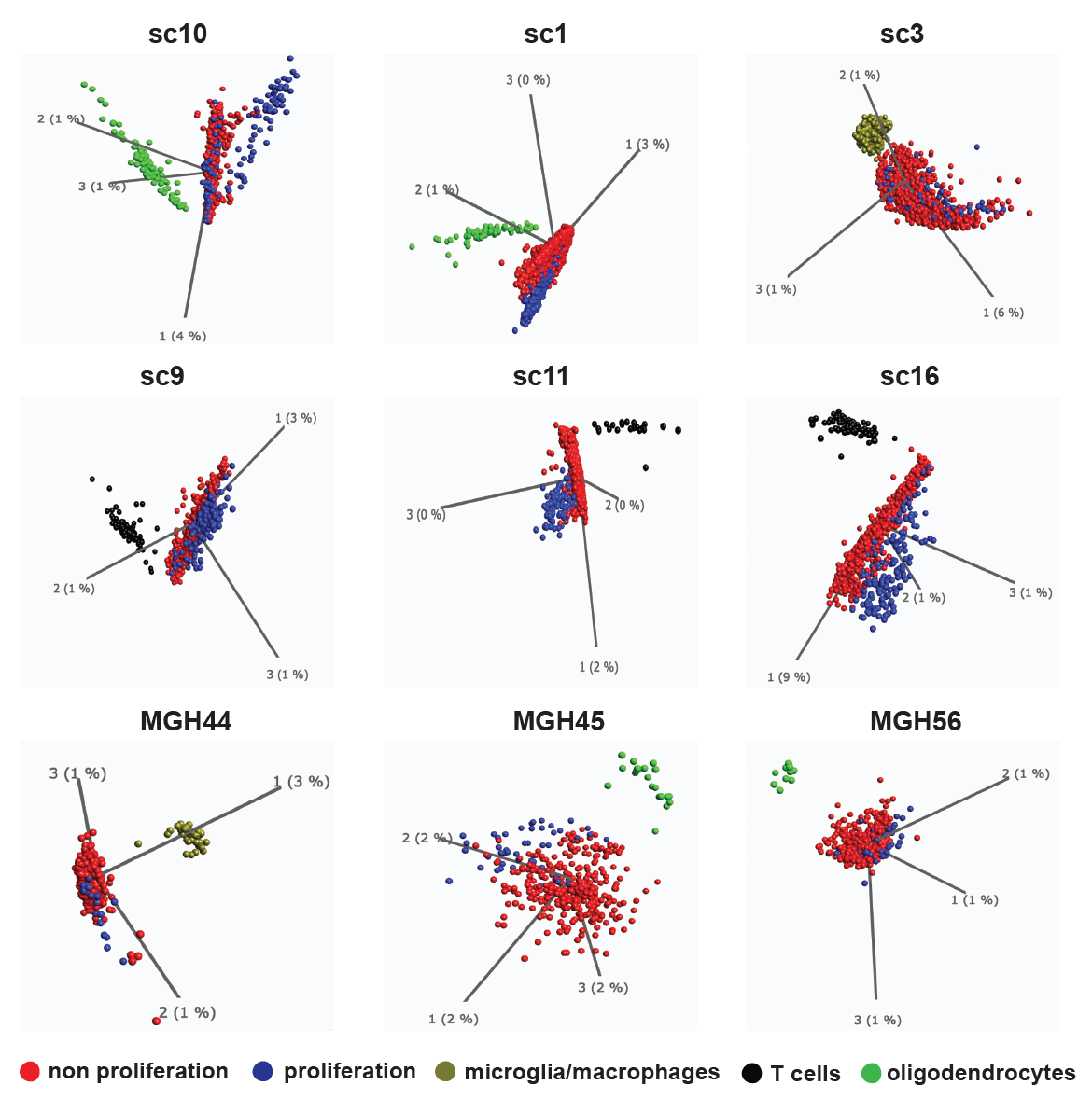
**Fig. S16. Proliferating and non-proliferating IDH-mutant glioma cells are similar at the global transcriptome level.**

With non-malignant cells as control, global transcriptome analyses were performed in 14 samples containing proliferating cells. PCA plots of 9 representative samples are shown.


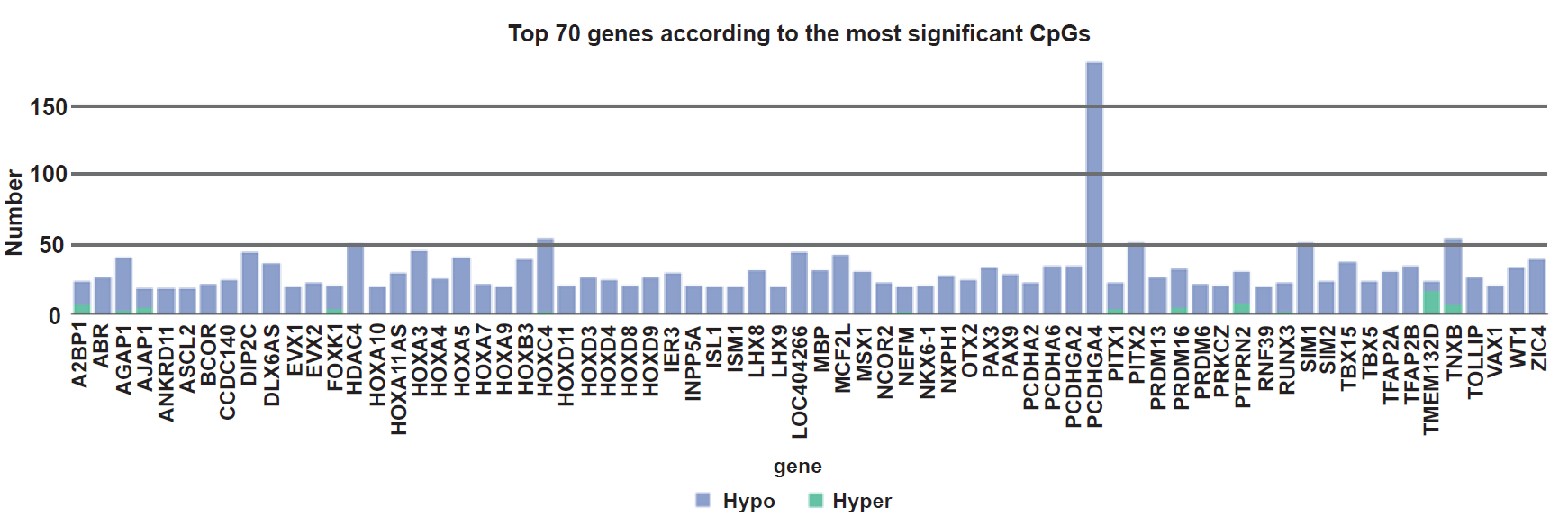
**Fig. S17. Top 70 genes with most significant CpGs in IDH-wildtype GBMs.**

Compared to normal brain tissue, the top 70 genes with most significant CpGs in IDH-wildtype GBM/EM samples are shown. Nealy all these genes showed hypomethylation. Hypo: hypomethylation, Hyper: hypermethylation.


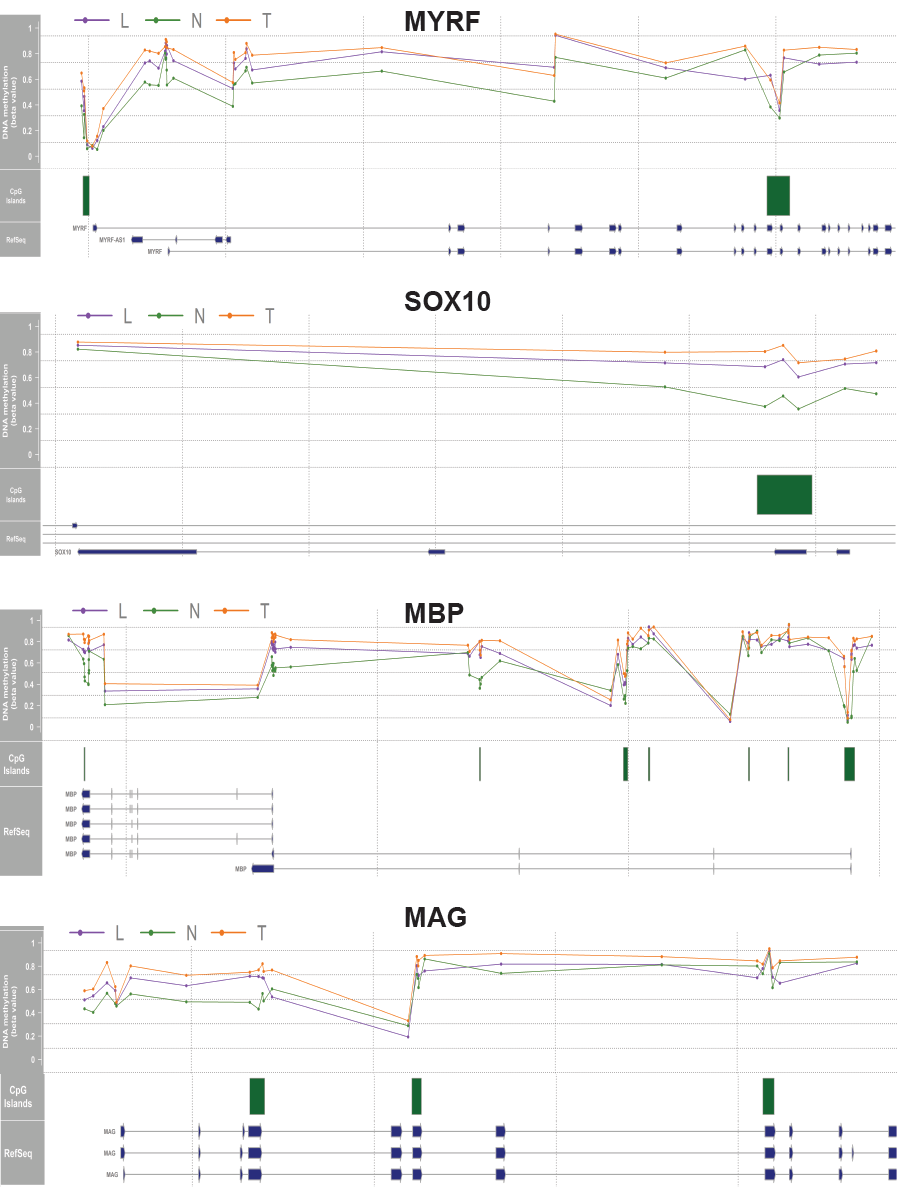


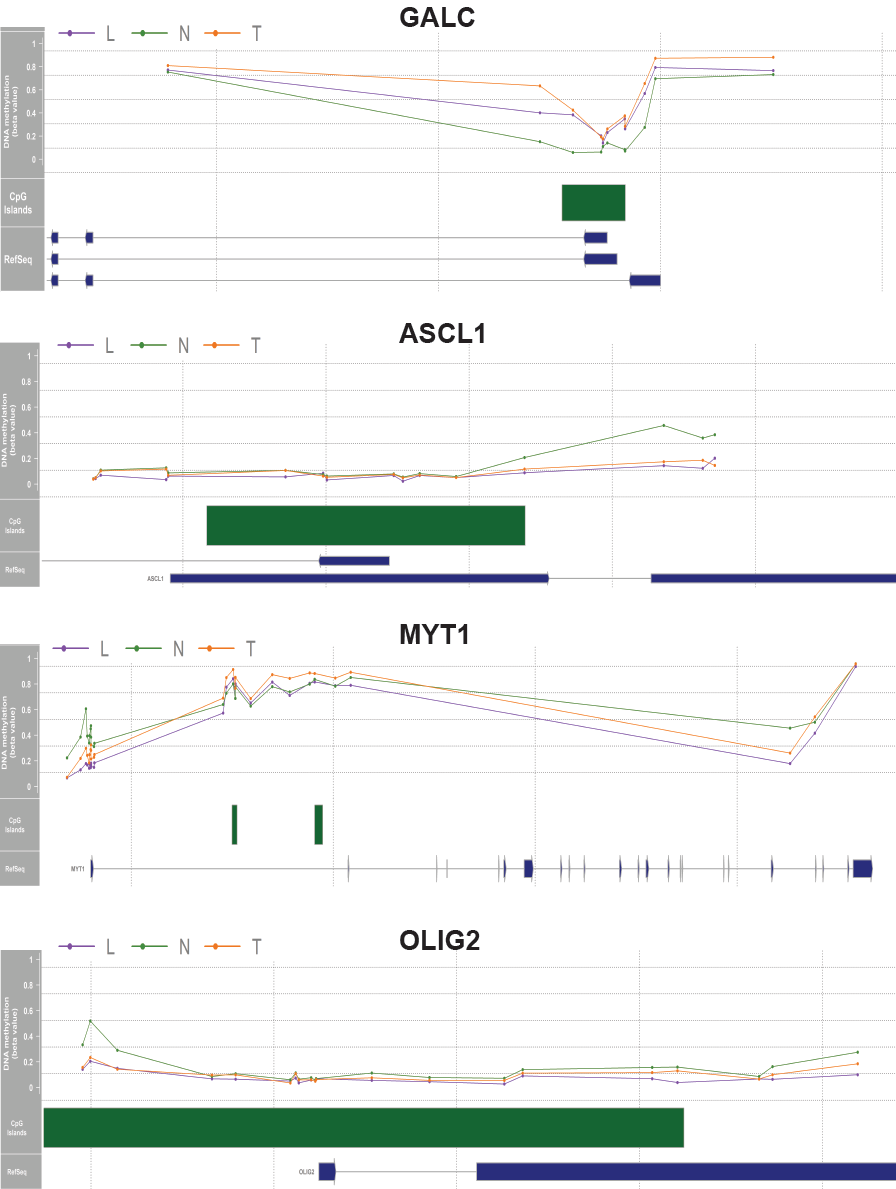


**Fig. S18. Myelination profiles of myelination regulator, hallmarks of MO and regulators of OPC specification and maintenance in PM gliomas.**

For each gene, beta values obtained from PM gliomas from TCGA (T), the local cohort (L) and normal brain tissues (N) (upper), and the corresponding transcripts (lower) are show.

**References**

1. Zhang Y, Chen K, Sloan SA, Bennett ML, Scholze AR, O'Keeffe S, Phatnani HP, Guarnieri P, Caneda C, Ruderisch N, et al: **An RNA-sequencing transcriptome and splicing database of glia, neurons, and vascular cells of the cerebral cortex.** *The Journal of neuroscience : the official journal of the Society for Neuroscience* 2014, **34:**11929-11947.

2. Kang HJ, Kawasawa YI, Cheng F, Zhu Y, Xu X, Li M, Sousa AM, Pletikos M, Meyer KA, Sedmak G, et al: **Spatio-temporal transcriptome of the human brain.** *Nature* 2011, **478:**483-489.

3. Marques S, van Bruggen D, Vanichkina DP, Floriddia EM, Munguba H, Varemo L, Giacomello S, Falcao AM, Meijer M, Bjorklund AK, et al: **Transcriptional Convergence of Oligodendrocyte Lineage Progenitors during Development.** *Dev Cell* 2018, **46:**504-517 e507.

4. Cahoy JD, Emery B, Kaushal A, Foo LC, Zamanian JL, Christopherson KS, Xing Y, Lubischer JL, Krieg PA, Krupenko SA, et al: **A transcriptome database for astrocytes, neurons, and oligodendrocytes: a new resource for understanding brain development and function.** *J Neurosci* 2008, **28:**264-278.

5. Marques S, Zeisel A, Codeluppi S, van Bruggen D, Mendanha Falcão A, Xiao L, Li H, Häring M, Hochgerner H, Romanov RA, et al: **Oligodendrocyte heterogeneity in the mouse juvenile and adult central nervous system.** *Science* 2016, **352:**1326-1329.
